# Dysregulation and prognostic potential of 5-methylcytosine (5mC), 5-hydroxymethylcytosine (5hmC), 5-formylcytosine (5fC), and 5-carboxylcytosine (5caC) levels in prostate cancer

**DOI:** 10.1186/s13148-018-0540-x

**Published:** 2018-08-07

**Authors:** Tine Maj Storebjerg, Siri H. Strand, Søren Høyer, Anne-Sofie Lynnerup, Michael Borre, Torben F. Ørntoft, Karina D. Sørensen

**Affiliations:** 1 Department of Urology, Aarhus University Hospital, Aarhus, Denmark; 2 Department of Pathology, Aarhus University Hospital, Aarhus, Denmark; 3 Department of Molecular Medicine, Aarhus University Hospital, Aarhus, Denmark

**Keywords:** Prostate cancer, Prognosis, Biomarker, Epigenetics, Immunohistochemistry, 5-methylcytosine, 5-hydroxymethylcytosine, 5-formylcytosine, 5-carboxylcytosine, ERG

## Abstract

**Background:**

Prognostic tools for prostate cancer (PC) are inadequate and new molecular biomarkers may improve risk stratification. The epigenetic mark 5-hydroxymethylcytosine (5hmC) has recently been proposed as a novel candidate prognostic biomarker in several malignancies including PC. 5hmC is an oxidized derivative of 5-methylcytosine (5mC) and can be further oxidized to 5-formylcytosine (5fC) and 5-carboxylcytosine (5caC). The present study is the first to investigate the biomarker potential in PC for all four DNA methylation marks in parallel. Thus, we determined 5mC, 5hmC, 5fC, and 5caC levels in non-malignant (NM) and PC tissue samples from a large radical prostatectomy (RP) patient cohort (*n* = 546) by immunohistochemical (IHC) analysis of serial sections of a tissue microarray. Possible associations between methylation marks, routine clinicopathological parameters, *ERG* status, and biochemical recurrence (BCR) after RP were investigated.

**Results:**

5mC and 5hmC levels were significantly reduced in PC compared to NM prostate tissue samples (*p* ≤ 0.027) due to a global loss of both marks specifically in *ERG−* PCs. 5fC levels were significantly increased in *ERG+* PCs (*p* = 0.004), whereas 5caC levels were elevated in both *ERG−* and *ERG+* PCs compared with NM prostate tissue samples (*p* ≤ 0.019). Positive correlations were observed between 5mC, 5fC, and 5caC levels in both NM and PC tissues (*p* < 0.001), while 5hmC levels were only weakly positively correlated to 5mC in the PC subset (*p* = 0.030). There were no significant associations between 5mC, 5fC, or *ERG* status and time to BCR in this RP cohort. In contrast, high 5hmC levels were associated with BCR in *ERG−* PCs (*p* = 0.043), while high 5caC levels were associated with favorable prognosis in *ERG+* PCs (*p* = 0.011) and were borderline significantly associated with worse prognosis in *ERG−* PCs (*p* = 0.058). Moreover, a combined high-5hmC/high-5caC score was a significant adverse predictor of post-operative BCR beyond routine clinicopathological variables in *ERG−* PCs (hazard ratio 3.18 (1.54–6.56), *p* = 0.002, multivariate Cox regression).

**Conclusions:**

This is the first comprehensive study of 5mC, 5hmC, 5fC, and 5caC levels in PC and the first report of a significant prognostic potential for 5caC in PC.

**Electronic supplementary material:**

The online version of this article (10.1186/s13148-018-0540-x) contains supplementary material, which is available to authorized users.

## Background

Prostate cancer (PC) is the most commonly diagnosed non-cutaneous malignancy among men in Europe and the USA [1], and approximately 1.6 million men were diagnosed worldwide in 2015 [2]. PC represents a heterogeneous group of cancers, ranging from indolent (clinically insignificant) to highly aggressive tumors with potential lethal outcome. Currently, serum prostate-specific antigen (PSA), Gleason score (GS), and TNM stage represent the best available prognostic tools for newly diagnosed PC, but are inadequate at predicting exact outcomes for individual patients. Novel prognostic biomarkers are needed to accurately identify aggressive PCs and focus active treatment (radical prostatectomy, RP) towards these patients, while avoiding unnecessary surgery and treatment-associated side effects in men with indolent PC.

The most frequently occurring genetic alterations in PC are genomic fusions between the Transmembrane protease, serine 2 (*TMPRSS2*) gene, and the ETS-related transcription factor gene (*ERG*), which are present in approximately half of all primary PCs and lead to *ERG* overexpression [3]. Several studies have investigated the prognostic value of *TMPRSS2:ERG* fusion status in early stage PC, but have shown conflicting results [4, 5]. In contrast, DNA methylation changes have shown promising prognostic potential [6–8].

Methylation on the 5-carbon position of cytosine (5-methylcytosine, 5mC) in CpG-dinucleotides is a well-characterized epigenetic mark involved in regulation of gene expression and chromatin structure. Cancer cells are characterized by aberrant hypermethylation of promoter-associated CpG islands, which is closely linked with transcriptional silencing of, e.g., tumor-suppressor genes, as well as by genome-wide DNA hypomethylation (i.e., global loss of 5mC) that is associated with chromosomal instability and activation of oncogenes [9–11].

Methylation of CpG-dinucleotides (5mC) is catalyzed by DNA methyltransferases (DNMTs) and can be erased by a family of α-ketoglutarate-dependent dioxygenases, named ten-eleven translocation (TET) proteins, through sequential oxidation of 5mC to 5-hydroxymethylcytosine (5hmC), 5-formylcytosine (5fC), and finally 5-carboxylcytosine (5caC) [12]. Subsequently, 5fC and 5caC are converted to unmethylated cytosine through base excision repair, completing the demethylation process [13–15].

Although tissue and cell-type specific variations occur, it has been estimated that ~ 5% of all cytosines in the genome of mammalian cells are marked as 5mC and less than 1% as 5hmC, while 5fC and 5caC are 10–1000-fold less abundant than 5hmC [12, 16]. Accordingly, it has been proposed that 5fC and 5caC may simply be short-lived intermediates in the active demethylation process, while 5hmC is likely to represent an independent epigenetic mark. Consistent with this, different chromatin-binding proteins have been shown to bind to 5mC and 5hmC, respectively, indicating distinct roles for 5mC and 5hmC in epigenomic regulation [17]. Some proteins, however, seem to bind specifically to 5fC or 5caC, suggesting possible independent epigenetic signaling functions for these marks as well [18, 19].

So far, the vast majority of epigenetic biomarker discovery studies have focused exclusively on 5mC and have not distinguished between 5mC and other less abundant DNA methylation marks. Yet, accumulating evidence suggests that global loss of 5hmC is an epigenetic hallmark of cancer, including PC [14, 20]. Indeed, a series of recent studies found reduced levels of 5hmC in glioma, colorectal, breast, liver, lung, pancreatic, and prostate cancer, as compared to corresponding normal tissues [14, 20, 21]. Furthermore, low 5hmC levels have been associated with poor outcome in glioma [22, 23], lung [24], cervical [25], breast [26], ovarian [27], and gastric [28] cancer, but with good prognosis in AML [29]. Moreover, by immunohistochemical (IHC) analysis of a tissue microarray (TMA) based on a large RP cohort, we recently demonstrated reduced 5hmC levels in *ERG* negative (*ERG−*) PCs [30]. We also observed that high 5hmC immunoreactivity was significantly associated with post-operative biochemical recurrence (BCR) in *ERG−* but not in *ERG* positive *(ERG+)* PCs [30]. Although 5hmC levels have been subject to increasing scrutiny in recent years, only one study has investigated the possible dysregulation of 5caC levels in cancer [21], and no previous studies have investigated 5fC nor 5caC in PC.

In the present study, we determined the global levels of all four DNA methylation marks (5mC, 5hmC, 5fC, and 5caC) in parallel, through IHC staining of serial sections of a large PC tissue microarray (TMA) [30] consisting of malignant cores from 546 RP patients, compared with > 300 matched adjacent non-malignant (NM) prostate tissue samples. We systematically investigated possible correlations between the four DNA methylation marks, *ERG* status, and routine clinicopathological parameters, and assessed the prognostic potential of each mark using post-operative BCR as clinical endpoint.

## Methods

### Tissue microarray

A TMA was generated using paraffin-embedded formalin-fixed RP tissue samples from 552 patients [30], who underwent curatively intended RP for histologically verified clinically localized PC at the Department of Urology, Aarhus University Hospital, Denmark, between 2001 and 2009. All PC specimens were re-graded by an expert uropathologist (SH) according to the 2014 International Society of Urological Pathology criteria for Gleason score [31].

Patients provided written informed consent and were followed passively until May 2015 with a mean/median clinical follow-up time of 82.9/80.0 months. By this time, six cases had withdrawn consent, leaving 546 PC patients eligible for IHC analysis on the TMA (for clinical characteristics see Table 1). For analyses of IHC scores (see below), another 88 patients were excluded because they had received either pre/post-operative endocrine or radiation treatment, had less than 3 months follow-up, or suffered BCR ≤ 3 months after RP. Details on the inclusion/exclusion process according to REMARK criteria are given in Additional files 1, 2, 3, 4, 5, and 6: Figures S1–S2, and clinicopathological data for the final patient sets used for biomarker evaluation are presented in Additional files 7, 8, 9, and 10: Tables S1A-D. The study was approved by the local scientific ethical committee and by the Danish Data Protection Agency.

**Table 1:** Clinical characteristics

	546 RP patients included on TMA	
Age at RP (years), median (range)	63 (34–76)	
Pathological GS		
< 7, *n* (%)	229 (41.9)	
≥ 7, *n* (%)	317 (58.1)	
Pathological T stage		
≤pT2c, *n* (%)	363 (66.5)	
≥pT3a, *n* (%)	182 (33.3)	
Unknown, *n* (%)	1 (0.2)	
Preoperative PSA		
PSA ≤ 10 ng/ml, *n* (%)	222 (40.7)	
PSA > 10 ng/ml, *n* (%)	324 (59.3)	
Surgical margin status		
Negative, *n* (%)	366 (67.0)	
Positive, *n* (%)	175 (32.1)	
Unknown, *n* (%)	5 (0.9)	
Follow-up (months), median (range)	80 (12–158)	
BCR		
No, *n* (%)	310 (56.8)	
Yes, *n* (%)	236 (43.2)	
	PC	NM
5mC (IHC), *n* (%)		
Total	344	328
Score < 1	–	3 (0.8)
Score = 1	160 (34.9)	126 (35.7)
Score > 1	184 (40.2)	199 (56.4)
Not determined	114 (24.9)	25 (7.1)
5hmC (IHC), *n* (%)		
Total	367	293
Score < 1	27 (5.9)	8 (2.5)
Score = 1	153 (33.4)	111 (34.0)
Score > 1	187 (40.8)	174 (53.4)
Not determined	91 (19.9)	33 (10.1)
5fC (IHC), *n* (%)		
Total	281	259
Score < 1	155 (33.8)	163 (50.6)
Score = 1	65 (14.2)	50 (15.5)
Score > 1	61 (13.3)	46 (14.3)
Not determined	177 (38.6)	63 (19.6)
5caC (IHC), *n* (%)		
Total	351	311
Score < 1	102 (22.2)	146 (41.4)
Score = 1	96 (21.0)	60 (17.0)
Score > 1	153 (33.4)	105 (29.7)
Not determined	107 (23.4)	42 (11.9)
*ERG* (IHC), *n* (%)		
Total	433	NA
*ERG−*	205 (44.8)	NA
*ERG+*	228 (49.8)	NA
Not determined	25 (5.4)	NA

Clinical data for the 546 RP patients analyzed on the TMA and distribution of IHC staining scores for each antibody in NM and PC tissue samples, respectively

*NA* not applicable

### IHC staining

Immunohistochemical staining for *ERG* and 5hmC has been previously described for this TMA [30]. Here, IHC staining for 5mC, 5fC, and 5caC was performed on serial sections of the same TMA, using the Benchmark XT fully automated stainer (Ventana). Slicing and heating was performed manually. TMA tissue sections (2.5 μm) were deparaffinized followed by endogenous peroxidase blocking using TBS/H_2_O_2_. Epitopes were retrieved using TEG pH 9.00 (5mC) or citrate pH 6.00 (5fC and 5caC) buffer. Subsequently, primary antibodies for 5mC, 5fC, and 5caC were applied (for details, see Additional file 11: Table S2). Secondary staining was performed with Horse Radish Peroxidase (HRP) conjugated rabbit secondary antibody (Envision, Cat. No. K4003, Dako), except for 5mC, which was detected by HRP conjugated mouse secondary antibody (Envision, Cat. No. K4001, Dako). Colorimetric signals were detected using diaminobenzidine (DAB), and sections were counterstained with hematoxylin for microscopic evaluation.

### IHC evaluation

**Fig. 1:**
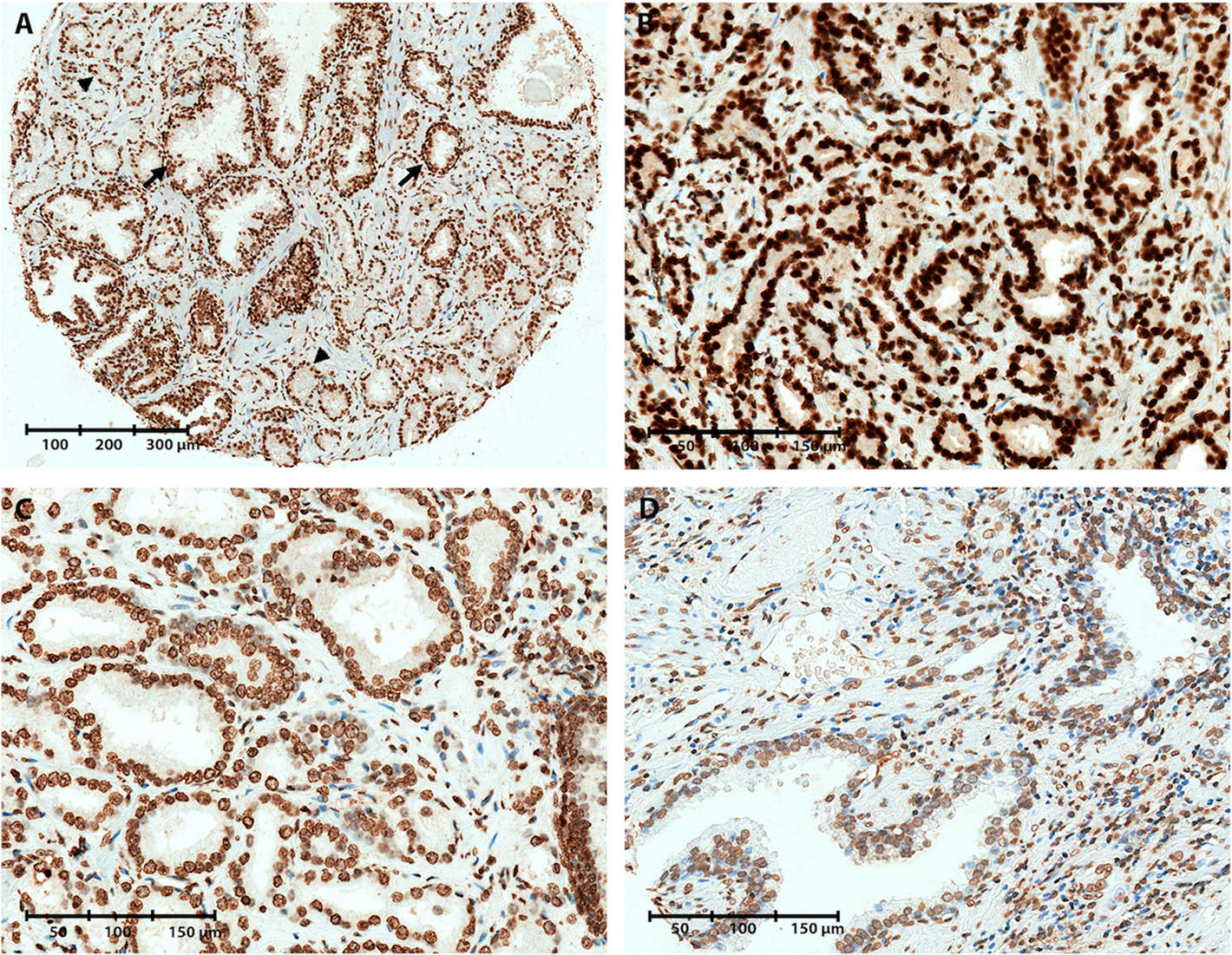
Representative images of 5mC immunoreactivity in malignant and non-malignant prostate tissue samples. **a** TMA core containing both malignant and NM prostate glands, illustrating reduced 5mC levels in malignant (IHC score = 1, arrowheads) compared to NM (IHC score = 2, arrows) glands. **b** Strong 5mC staining in a malignant core (IHC score = 2). **c** Moderate 5mC staining in a malignant core (IHC score = 1). **d** Weak 5mC staining in a NM core (IHC score = 0)

**Fig. 2:**
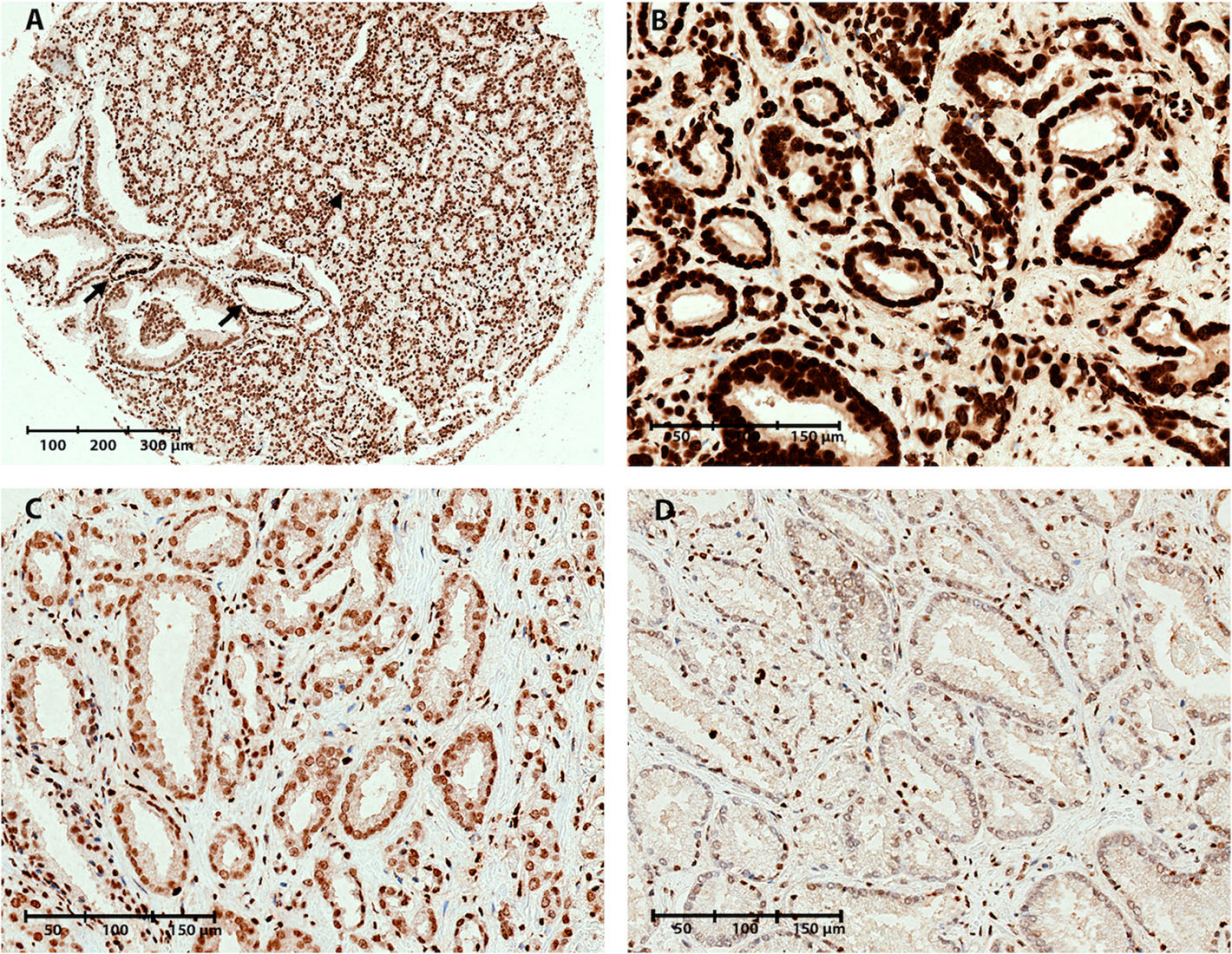
Representative images of 5hmC immunoreactivity in malignant and non-malignant prostate tissue samples. **a** TMA core containing both malignant and NM prostate glands, illustrating reduced 5hmC levels in malignant (IHC score = 1, arrowheads) compared to NM glands (IHC score = 2, arrows). **b** Strong 5hmC staining in a malignant core (IHC score = 2). **c** Moderate 5hmC staining in a malignant core (IHC score = 1). **d** Weak 5hmC staining in malignant core (IHC score = 0)

**Fig. 3:**
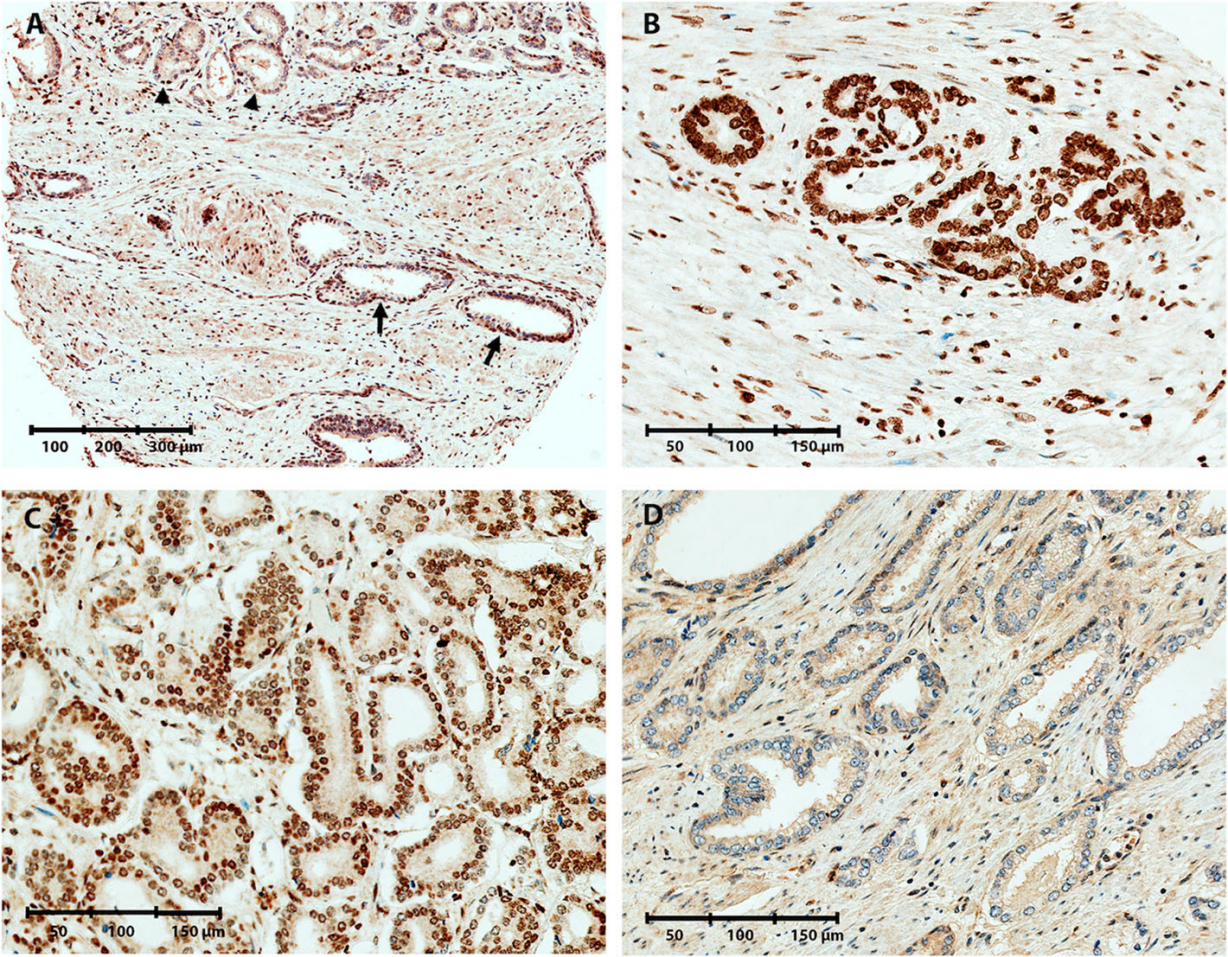
Representative images of 5fC immunoreactivity in malignant and non-malignant prostate tissue samples. **a** TMA core containing both malignant (IHC score = 0, arrowheads), and NM (IHC score = 0, arrows) glands. **b** Strong 5fC staining in a malignant core (IHC score = 2). **c** Moderate 5fC staining in a malignant core (IHC score = 1). **d** Weak 5fC staining in a malignant core (IHC score = 0)

**Fig. 4:**
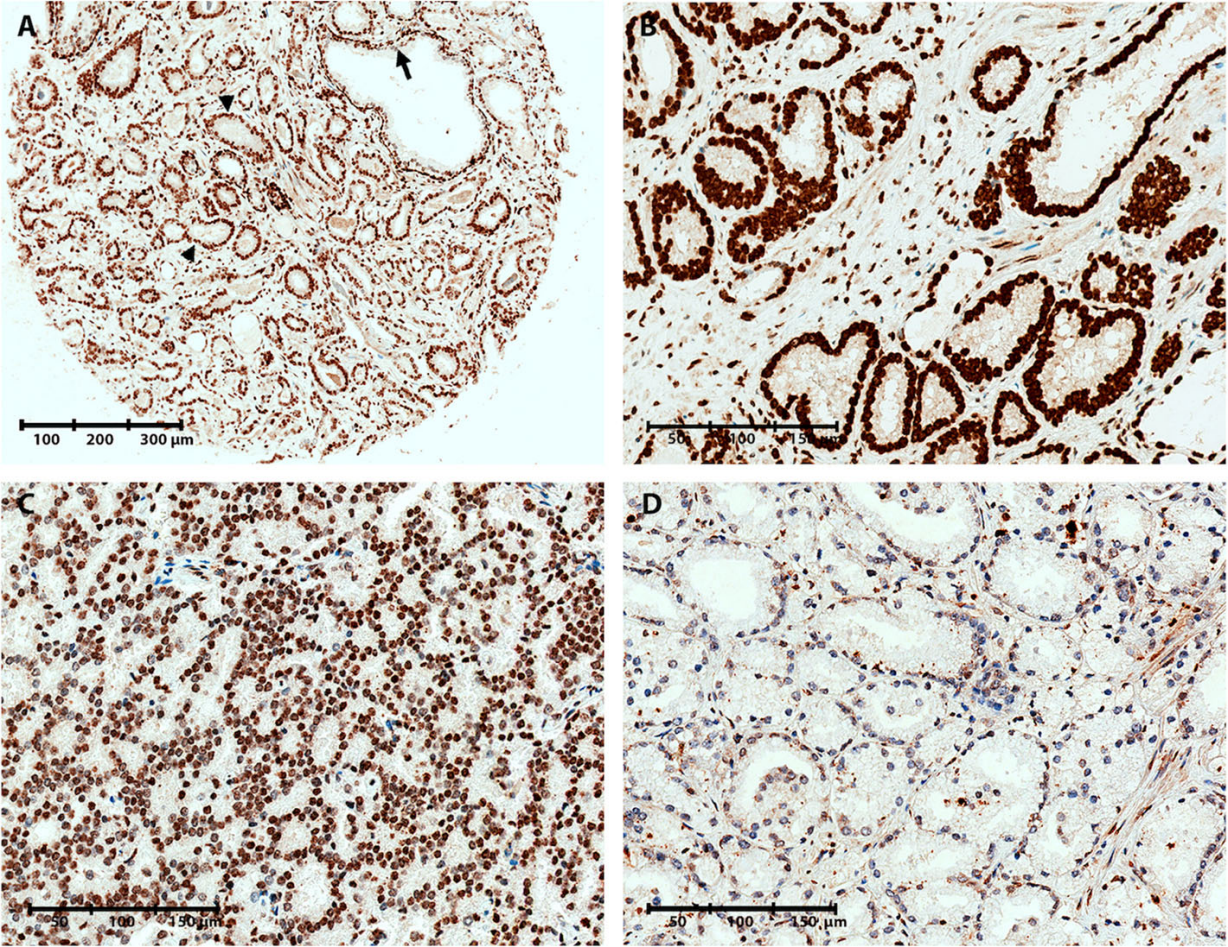
Representative images of 5caC immunoreactivity in malignant and non-malignant prostate tissue samples. **a** TMA core containing both malignant and NM prostate glands, illustrating increased 5caC levels in malignant (IHC score = 2, arrowheads) compared to NM glands (IHC score = 0, arrow). **b** Strong 5caC staining in a malignant core (IHC score = 2). **c** Moderate 5caC staining in a malignant core (IHC score = 1). **d** Weak 5caC staining in a malignant core (IHC score = 0)

Immunoreactivity for 5mC, 5fC, and 5caC was evaluated by two independent observers (SH and TMS) using the Pannoramic Viewer software (3DHISTECH, Hungary). A numerical IHC score was given for each core, based on the antibody staining intensity in the nuclei of malignant or NM prostate epithelial cells, respectively (0, no-weak; 1, moderate; 2, strong; see Figs. 1, 2, 3, and 4 for representative images). As some cores had changed status from malignant to NM or vice-versa from one TMA section to the next, we carefully re-evaluated the PC/NM status of every core during IHC scoring. In addition, some cores were lost during TMA processing. In total, malignant cores from 344, 367, 281, and 351 PC patients could be evaluated for 5mC, 5hmC, 5fC, and 5caC immunoreactivity, respectively (Additional file 1: Figure S1; Additional file 7, 8, 9, and 10: Tables S1A–D). NM tissue samples could be evaluated from 328 (5mC), 293 (5hmC), 259 (5fC), and 311 (5caC) patients in total (Additional file 2: Figure S2).

For 5hmC and *ERG*, we used immunohistochemistry scores from neighboring sections of the exact same TMA from a previous study [30]. In the present study, all patients for whom at least one malignant or at least one NM core could be evaluated for each methylation mark were included in the final analyses, which for 5hmC resulted in a moderately larger patient set (*n* = 367) than in our previous work (*n* = 311) [30]. For patients with multiple PC/NM cores that could be evaluated for each antibody, we calculated a mean IHC score for each tissue type for each patient (< 1, weak; = 1, moderate; > 1, strong). Positive *ERG* immunoreactivity was used as a proxy for *ERG* fusion status [32]. As described previously [30], a PC patient was considered *ERG+* if nuclear *ERG* immunoreactivity was detected in at least one malignant core from that patient, and otherwise as *ERG−*.

### Statistical methods

All statistical analyses were performed using Stata IC version 14 (StataCorp, College Station TX, USA). *p* values < 0.05 were considered significant. Associations between methylation marks and clinicopathological characteristics were evaluated by two-sided chi^2^ tests. Spearman’s rank correlation coefficients were used to assess correlations between 5mC, 5hmC, 5fC, and 5caC levels in patients where all four marks could be evaluated.

Uni- and multivariate Cox regression analyses and Kaplan-Meier analyses were used to test the prognostic value of *ERG*, 5mC, 5hmC, 5fC, and 5caC, using BCR (defined as PSA ≥ 0.2 ng/ml in two consecutive measurements after RP) as clinical endpoint. For recurrence-free survival analyses, patients were censored at their last clinical follow-up. Statistical significance in Kaplan-Meier analysis was evaluated using 2-sided log-rank tests. Predictive accuracy was estimated using Harrell’s C-index [33].

For analysis of methylation marks as dichotomized variables, we used mean IHC score ≤ 1 vs. > 1 as cut-point. To evaluate the prognostic value of a combination of 5hmC score and 5caC score, we divided patients into three subgroups: low (5hmC ≤ 1 and 5caC ≤ 1), moderate (5hmC ≤ 1 and 5caC > 1, or 5hmC > 1 and 5caC ≤ 1), and high (5hmC > 1 and 5caC > 1).

## Results

### Methylation levels in PC compared with NM specimens

By IHC staining of a TMA from a large RP cohort (*n* = 546; Table 1), we assessed the levels of DNA methylation marks 5mC, 5hmC, 5fC, and 5caC. Nuclear staining intensity in NM and PC epithelial cells, respectively, was scored as 0 (weak staining), 1 (moderate staining), or 2 (strong staining) (Figs. 1, 2, 3, and 4).

**Fig. 5:**
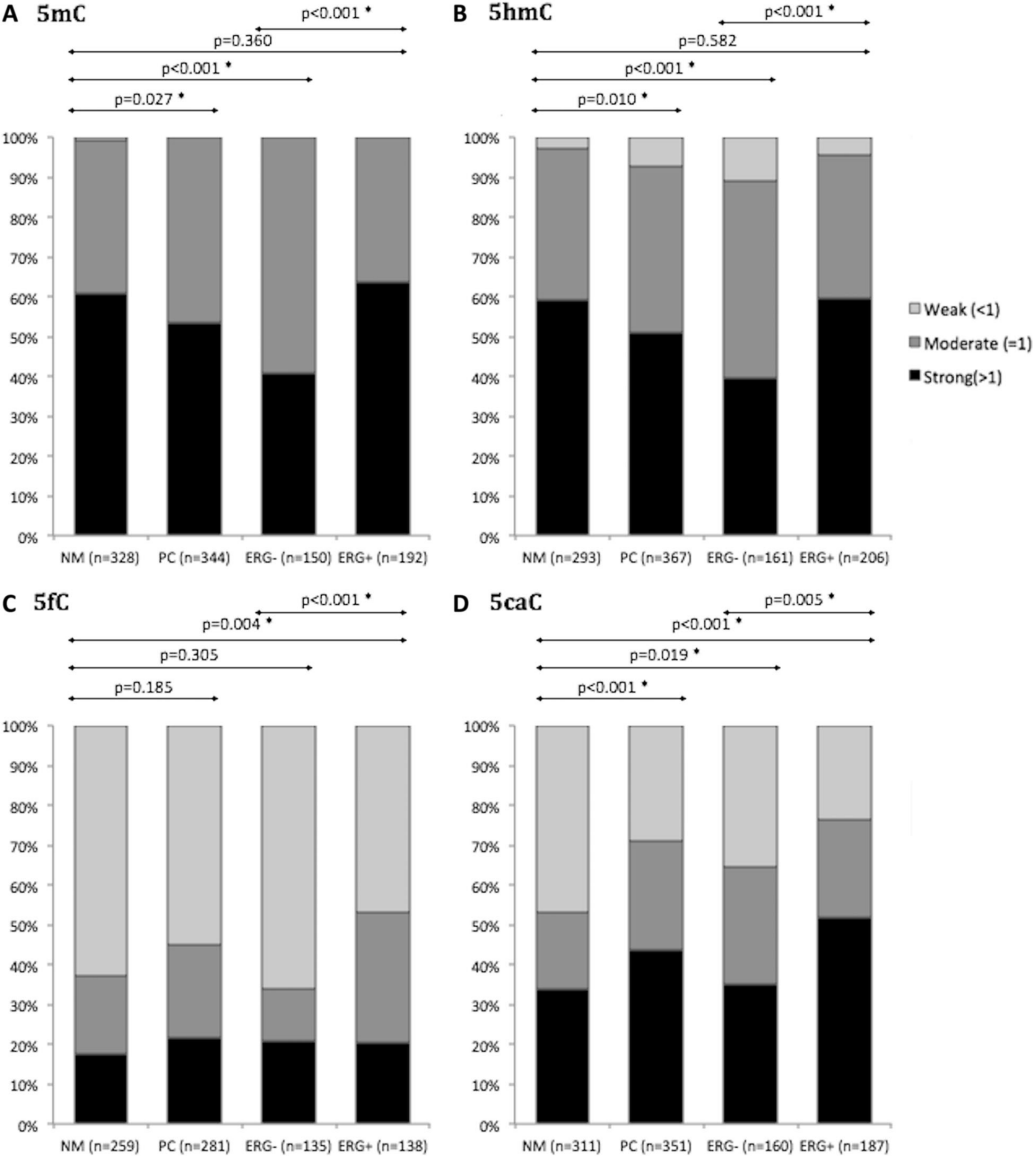
Distribution of methylation mark IHC scores in PC and NM tissue samples. Histograms show the distribution of samples with strong (score > 1; black boxes), moderate (score = 1; dark gray boxes), or weak (score < 1; light gray boxes) immunoreactivity for each methylation mark. **a** Distribution of 5mC scores in NM and PC samples from the full cohort, and in *ERG−* and *ERG+* PC samples (two patients had unknown *ERG* status). **b** Distribution of 5hmC scores in NM and PC samples from the full cohort, and in *ERG−* and *ERG+* PC samples. **c** Distribution of 5fC scores in NM and PC samples from the full cohort, and in *ERG−* and *ERG+* PC samples (eight patients had unknown *ERG* status). **d** Distribution of 5caC scores in NM and PC samples from the full cohort, and in *ERG−* and *ERG+* PC samples (four patients had unknown *ERG* status). Significant *p* values (chi^2^ test) are marked by an asterisk (*)

Staining for 5mC could be evaluated in 344 PC and 328 NM specimens (Table 1). We found that 5mC levels were moderately, but statistically significantly reduced in PC compared with NM tissue samples (*p* = 0.027; chi^2^ test; Fig. 5a). The reduction in 5mC levels was specific for *ERG−* PCs (*p* < 0.001; chi^2^ test; Fig. 5a), while 5mC levels were similar in *ERG+* PCs and NM samples (*p* = 0.360; chi^2^ test; Fig. 5a). The significant decrease of 5mC staining observed in *ERG−* PC (Fig. 5a) was also confirmed by a paired analysis of 107 patients for whom matched NM and *ERG−* PC samples with 5mC score were available (data not shown). Specifically, 38% (41/107) of these patients had an altered 5mC score in the *ERG−* PC sample, most of which were reduced (71%; 29/41 patients) compared to the matched NM sample.

Staining for 5hmC could be evaluated in 367 PC and 293 NM specimens (Table 1). For the full RP cohort, we observed a significant reduction of 5hmC staining in PC compared with NM tissue samples (*p* = 0.010; chi^2^ test; Fig. 5b). This was explained by a global loss of 5hmC specifically in *ERG−* PCs (*p* < 0.001; chi^2^ test; Fig. 5b), whereas 5hmC levels were similar in *ERG+* PC and NM samples (*p* = 0.582; chi^2^ test; Fig. 5b), consistent with our previous report [30]. The significant decrease of 5hmC staining observed in *ERG−* PC (Fig. 5b) was also corroborated by a paired analysis of 107 patients for whom matched NM and *ERG−* PC samples with 5hmC score were available. Here, 42% (45/107) displayed an altered 5hmC score in the *ERG−* PC sample, the majority of which were reduced (78%; 35/45 patients) as compared to the matched NM sample.

5fC levels could be evaluated in 281 PC and 259 NM specimens (Table 1). There were no significant differences in 5fC levels in the full PC set compared with NM tissue samples (*p* = 0.185; chi^2^ test; Fig. 5c), nor in *ERG-* PCs compared with NM samples (*p* = 0.305; chi^2^ test; Fig. 5c). However, *ERG+* PCs displayed significantly higher 5fC scores than NM prostate tissue samples (*p* = 0.004; chi^2^ test; Fig. 5c). A similar pattern was seen in a paired analysis of 83 patients for whom matched NM and *ERG+* PC samples with 5fC score were available (data not shown). Specifically, 47% (39/83) of these patients had a changed 5fC score in the *ERG+* PC sample, most of which went up (54%; 21/39 patients) as compared to the matched NM sample, together also indicating some interpatient variation for 5fC.

Finally, 5caC immunoreactivity could be evaluated in 351 PC and 311 NM specimens (Table 1) and was significantly stronger in PC tissue samples (*p* < 0.001; chi^2^ test; Fig. 5d). Elevated 5caC levels were seen both in *ERG−* PCs (*p* = 0.019; chi^2^ test; Fig. 5d) and was even more pronounced in *ERG+* PCs (*p* < 0.001; chi^2^ test; Fig. 5d), as compared to NM samples. This was confirmed in a paired analysis of 113 patients for whom matched NM and *ERG−* PC samples with 5caC scores were available (data not shown). Here, 46% (52/113) of the patients had an altered 5caC score in the matched *ERG−* PC sample, the majority of which were increased (65%; 34/52 patients). Likewise, a paired analysis of 128 patients for whom matched NM and *ERG+* PC samples with 5caC scores were available (data not shown) showed that 52% (66/128) of the patients had an altered 5caC score in the paired *ERG+* PC sample, most of which went up (79%; 52/66 patients).

In summary, we observed a significant reduction of 5mC and 5hmC levels in *ERG−* but not in *ERG+* PCs, compared to NM prostate tissue samples. In addition, 5fC levels were moderately increased in *ERG+* but not in *ERG−* PCs, whereas 5caC levels were elevated in both *ERG−* and *ERG+* PCs. These findings were also corroborated by paired analyses of the subset of patients for whom matched NM and PC samples could be evaluated and scored.

Furthermore, based on immunoreactivity scores for 232 PC and 209 NM specimens that could be evaluated for all four DNA methylation marks, we observed moderate positive correlations between 5mC, 5fC, and 5caC levels in both PC and NM samples (Spearman’s correlations: rho 0.43–0.53, *p* < 0.001; Additional file 12: Table S3). In contrast, 5hmC levels were weakly positively correlated only to 5mC and only in the PC subset (rho 0.14; *p* = 0.030; Additional file 12: Table S3). These results are consistent with previous reports, suggesting that 5hmC is an independent epigenetic mark [17, 34, 35].

### Association of 5mC, 5hmC, 5fC, and 5caC levels with clinicopathological parameters

Next, we investigated possible correlations between IHC scores for the four DNA methylation marks in PC tissue samples and key clinicopathological parameters associated with tumor aggressiveness, i.e., GS, pathological tumor (pT) stage, preoperative PSA level, surgical margin (SM) status, and BCR status.

5mC levels were not significantly associated with any of the clinicopathological parameters in the full PC patient set (*p* ≥ 0.122; chi^2^ test; Additional file 3: Figure S3A). We also found no significant correlations in *ERG−* (*p* ≥ 0.495; Additional file 3: Figure S3B) or in *ERG+* PCs (*p* ≥ 0.150, Additional file 3: Figure S3C), except that a higher 5mC score was weakly associated with higher GS in the *ERG+* PC subgroup (*p* = 0.045; Additional file 3: Figure S3C).

Strong 5hmC staining was significantly associated with post-operative BCR in the full PC cohort (*p* = 0.038; Additional file 4: Figure S4A), but not with any of the routine clinicopathological variables (*p* ≥ 0.317; Additional file 4: Figure S4A). Similarly, in *ERG−* PCs, strong 5hmC staining was associated with BCR (*p* = 0.015) and advanced pT stage (*p* = 0.001) (Additional file 4: Figure S4B). There were no other significant correlations in the *ERG−* nor in the *ERG+* subset (*p* ≥ 0.146; chi^2^ test; Additional file 4: Figure S4B, C). In summary, high 5hmC levels were associated with adverse clinical parameters in *ERG−* PCs, consistent with our previous findings for a smaller subset of this cohort [30].

**Fig. 6:**
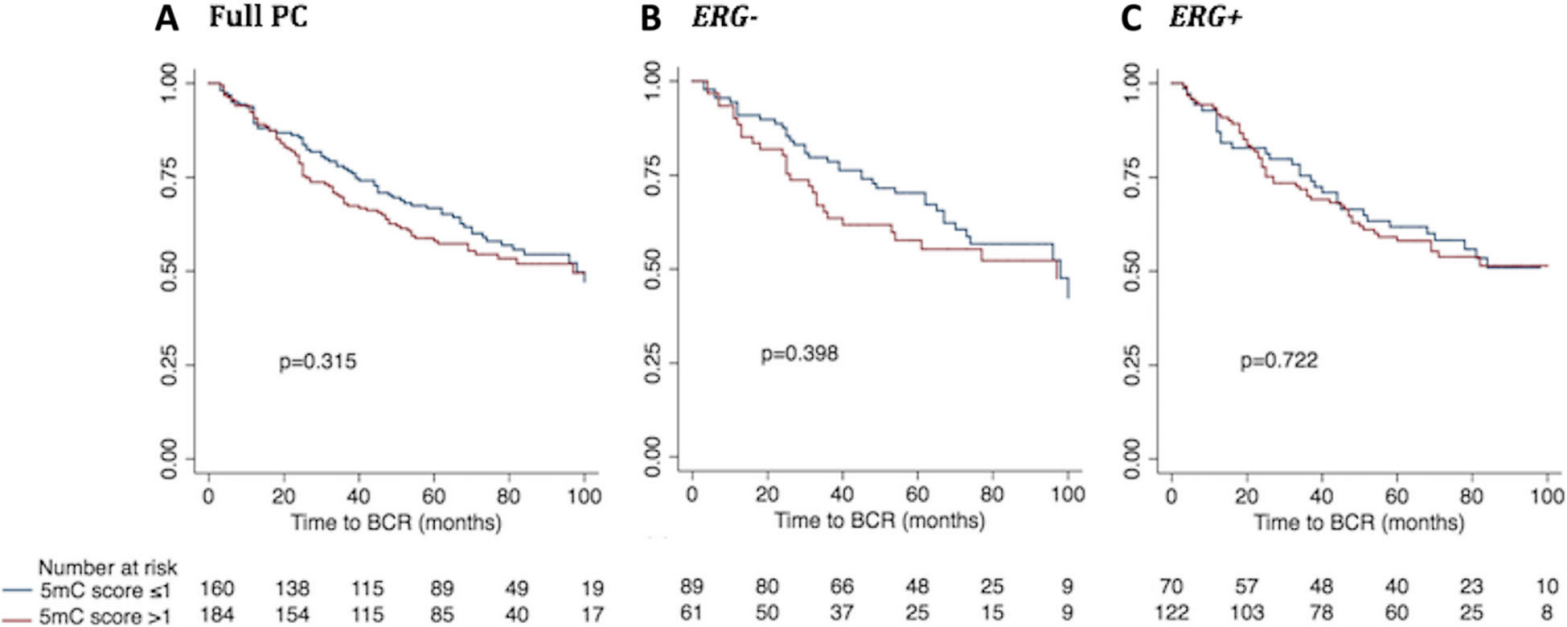
Kaplan-Meier analysis: Association between 5mC score and time to BCR after RP. The prognostic value of 5mC score was evaluated through Kaplan-Meier analysis using time to BCR after RP as the clinical endpoint. Patients with low 5mC score (≤ 1; blue curves) were compared with patients with high 5mC score (> 1; red curves) in (**a**) the full PC patient set, **b** the *ERG−* PC subset, and **c** the *ERG+* PC subset. The number of patients in each subgroup are listed at the bottom and *p* values for 2-sided log-rank tests are given for each panel. No significant differences were observed

In the full PC set, as well as in the *ERG+* PC subset, stronger 5fC staining was significantly associated with low pT stage (<pT3; *p* = 0.023/*p* = 0.019) and low BCR risk (*p* = 0.049/*p* = 0.019), but not with any other clinical parameters (Additional file 5: Figure S5A and C). In contrast, there were no significant correlations in *ERG−* PCs (*p* ≥ 0.227; Fig. 6b). Thus, high 5fC levels may be associated with favorable prognosis in *ERG+* PCs.

In the full PC set, stronger 5caC staining was significantly associated with low pT stage (*p* = 0.004) and low pre-operative PSA (*p* = 0.019; Additional file 6: Figure S6A). Similarly, in *ERG+* PCs, stronger 5caC staining was associated with low pre-operative PSA (*p* = 0.006), low pT stage (*p* < 0.001), and low BCR risk (*p* = 0.019; Additional file 6: Figure S6C). However, in *ERG−* PCs, stronger 5caC staining was associated with higher GS (*p* = 0.012; Additional file 6: Figure S6B). These results suggest that high 5caC levels may be associated with less aggressive disease in *ERG+* PC, but with more aggressive disease in *ERG−* PCs.

### Prognostic value of DNA methylation levels in PC

To evaluate the potential prognostic value of 5mC, 5hmC, 5fC, and 5caC levels, we used time to BCR after RP as the clinical endpoint.

In the full RP cohort, there were no significant correlations between 5mC immunoreactivity in PC tissue samples and time to BCR in univariate Cox regression analysis (5mC *continuous/dichotomized* variable: *p* = 0.834/*p* = 0.318; Table 2) or in Kaplan-Meier analysis (*p* = 0.315; Fig. 6a). 5mC staining was also not significantly associated with time to BCR in the *ERG−* or *ERG+* subgroup in Cox regression analysis (*p* ≥ 0.401; Table 2) or in Kaplan-Meier analysis (*p* ≥ 0.398; Fig. 6b, c). All established clinicopathological prognostic parameters (high PSA, high GS, positive SM status, and advanced pT stage) were significantly associated with shorter time to BCR in this RP patient set (*p* < 0.001, Additional file 13: Table S4), indicating that it is a representative cohort. Furthermore, *ERG* status did not predict time to BCR in our RP cohort (*p* = 0.840; Additional file 13: Table S4), consistent with other studies [36, 37].

**Table 2:** Univariate Cox regression analysis of BCR-free survival for 5mC, 5hmC, 5fC, and 5caC score

Variable	Full PC patient set			*ERG-* PC patient subset			*ERG+* PC patient set		
	HR (95% CI)	*p* value	C-index	HR (95% CI)	*p* value	C-index	HR (95% CI)	*p* value	C-index
5mC score (cont.)	1.04 (0.72–1.50)	0.834	0.51	1.26 (0.69–2.28)	0.454	0.54	0.88 (0.54–1.43)	0.599	0.52
5mC score (dich.)	1.18 (0.85–1.62)	0.318	0.53	1.23 (0.76–2.00)	0.401	0.54	1.08 (0.70–1.69)	0.723	0.51
5hmC score (cont.)	1.37 (1.01–1.87)	*0.045*	0.55	1.62 (1.02–2.57)	*0.043*	0.59	1.14 (0.75–1.75)	0.537	0.51
5hmC score (dich.)	1.40 (1.02–1.92)	*0.038*	0.55	1.93 (1.19–3.14)	*0.008*	0.60	1.05 (0.69–1.61)	0.805	0.50
5fC score (cont.)	0.94 (0.73–1.20)	0.613	0.51	1.10 (0.78–1.56)	0.583	0.53	0.75 (0.52–1.09)	0.130	0.55
5fC score (dich.)	1.02 (0.67–1.56)	0.926	0.50	1.38 (0.77–2.49)	0.282	0.54	0.67 (0.34–1.33)	0.253	0.53
5caC score (cont.)	0.91 (0.73–1.14)	0.419	0.53	1.21 (0.86–1.71)	0.277	0.54	0.68 (0.51–0.92)	*0.011*	0.60
5caC score (dich.)	1.01 (0.73–1.40)	0.966	0.49	1.61 (0.98–2.63)	0.058	0.56	0.62 (0.40–0.97)	*0.034*	0.57

Significant *p* values are highlighted in italics

*HR* hazard ratio, *CI* confidence interval, *C-index* Harrell’s C-index

**Fig. 7:**
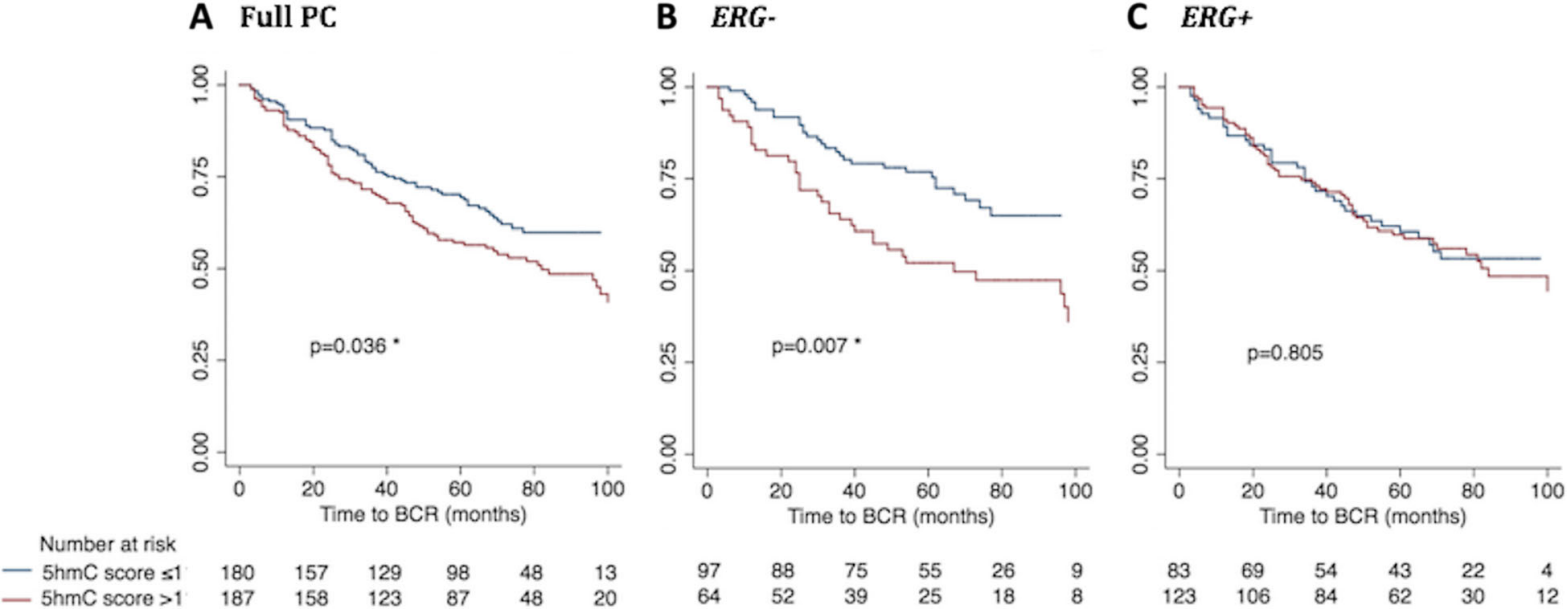
Kaplan-Meier analysis: Association between 5hmC score and time to BCR after RP. The prognostic value of 5hmC score was evaluated through Kaplan-Meier analysis using time to biochemical recurrence after radical prostatectomy as the clinical endpoint. Patients with low 5hmC score (≤ 1; blue curves) were compared with patients with high 5hmC score (> 1; red curves) in (**a**) the full PC patient set, **b** the *ERG−* PC subset, and **c** the *ERG+* PC subset. The number of patients in each subgroup is listed at the bottom and *p* values for 2-sided log-rank tests are given for each panel. Significant *p* values are marked by an asterisk (*)

In accordance with our previous work [30], high 5hmC levels were significantly associated with shorter BCR time in the full PC patient set in univariate (continuous: *p* = 0.045; Table 2) and multivariate Cox regression analysis (continuous: *p* = 0.026; Additional file 14: Table S5), as well as in Kaplan-Meier analysis (*p* = 0.036; log-rank test; Fig. 7a). The significant association was specific to the *ERG−* PC subgroup, where high 5hmC scores were significantly associated with BCR in both univariate (*p* = 0.043; Table 2) and multivariate Cox regression analysis (*p* = 0.042; Additional file 14: Table S5) as well as in Kaplan-Meier analysis (*p* = 0.007; Fig. 7b), but not in *ERG+* PCs (*p* = 0.537; Table 2; *p* = 0.805; Fig. 7c). We obtained similar results when 5hmC was analyzed as a dichotomized variable by Cox regression in both the full PC set and in the *ERG* stratified subsets (Table 2 and Additional file 15: Table S6).

**Fig. 8:**
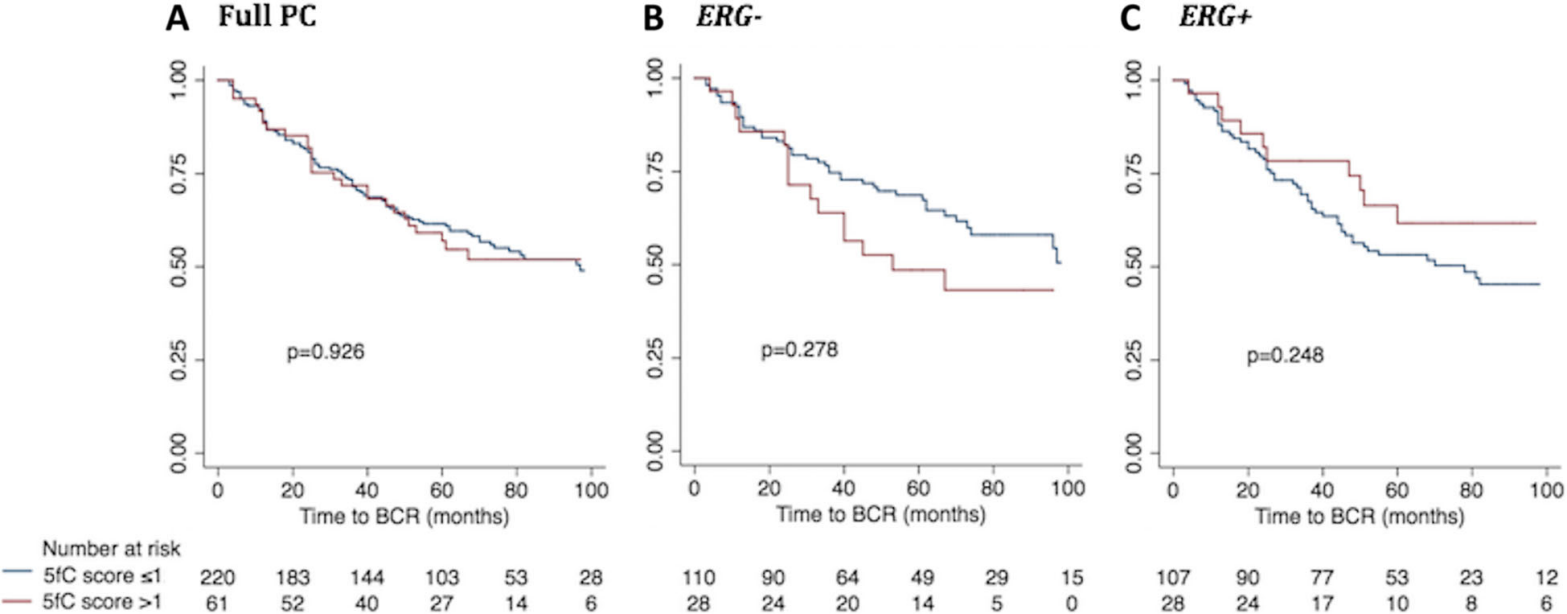
Kaplan-Meier analysis: Association between 5fC score and time to BCR after RP. The prognostic value of 5fC score was evaluated through Kaplan-Meier analysis using time to biochemical recurrence after radical prostatectomy as the clinical endpoint. Patients with low 5fC score (≤ 1; blue curves) were compared with patients with high 5fC score (> 1; red curves) in (**a**) the full PC patient set, **b** the *ERG−* PC subset, and **c** the *ERG+* PC subset. The number of patients in each subgroup is listed at the bottom and *p* values for 2-sided log-rank tests are given for each panel. No significant differences were observed

For 5fC, we observed no significant correlation with time to BCR in univariate Cox regression analysis (5fC continuous/dichotomized: *p* = 0.613/*p* = 0.926, Table 2) or in Kaplan Meier analysis (*p* = 0.926; Fig. 8a) in the full PC patient set, and also not after stratification for *ERG* status (*ERG−/ERG+: p* ≥ 0.282/*p* ≥ 0.130, Additional file 11: Table S2; and *p* = 0.278/*p* = 0.248, Fig. 8b, c). Accordingly, multivariate Cox regression analyses were not performed for 5fC (Additional file 16: Table S7).

**Fig. 9:**
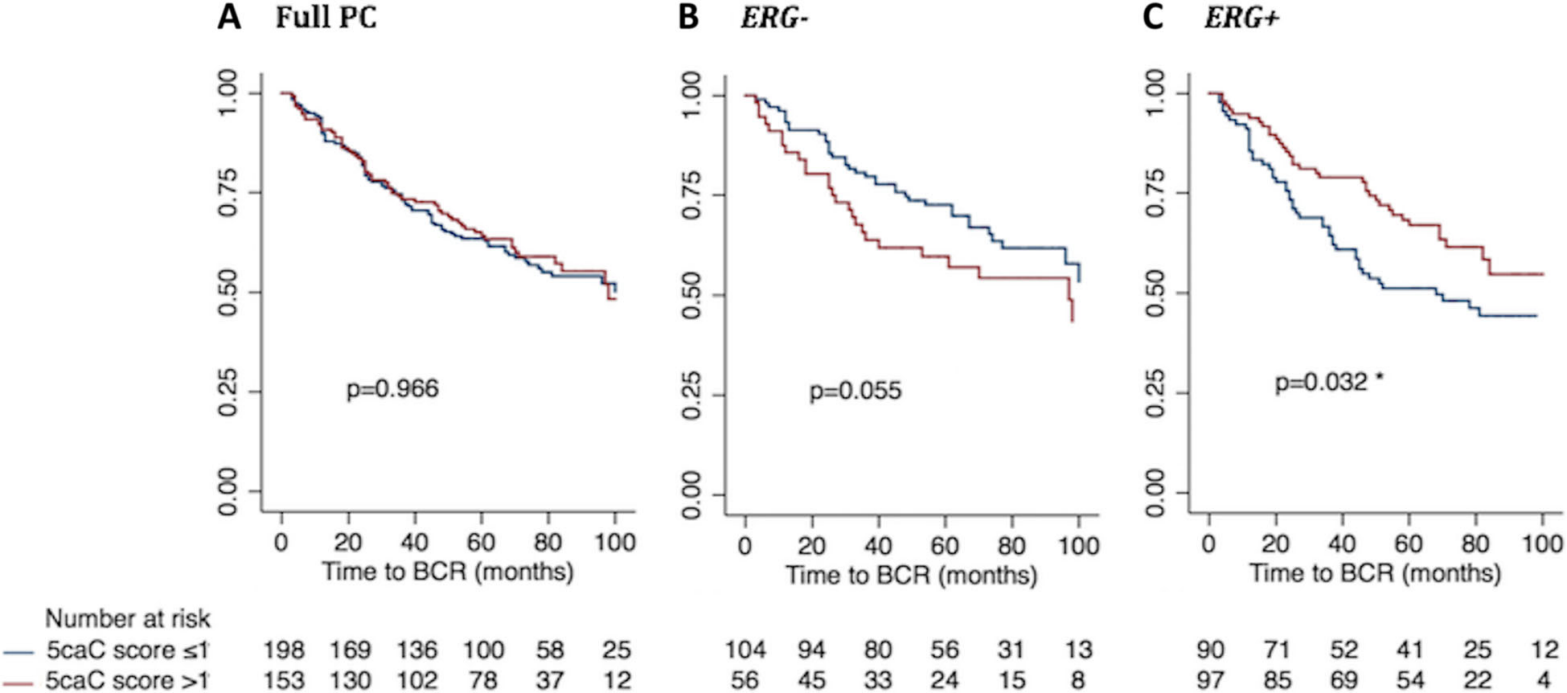
Kaplan-Meier analysis: Association between 5caC score and time to BCR after RP. The prognostic value of 5caC score was evaluated through Kaplan-Meier analysis using time to biochemical recurrence after radical prostatectomy as the clinical endpoint. Patients with low 5caC score (≤ 1; blue curves) were compared with patients with high 5caC score (> 1; red curves) in (**a**) the full PC set, **b** the *ERG−* PC subset, and **c** the *ERG+* PC subset. The number of patients in each subgroup is listed at the bottom and *p* values for 2 -sided log-rank tests are given for each panel. Significant *p* values are marked by an asterisk (*)

For 5caC, we found no significant correlation with time to BCR in the full patient set in univariate Cox regression analysis (continuous/dichotomized: *p* = 0.419/*p* = 0.966; Table 2) or in Kaplan-Meier analysis (*p* = 0.966; Fig. 9a). Likewise, in *ERG−* PCs, there was no significant correlation between time to BCR when 5caC score was analyzed as a continuous variable (*p* = 0.277; Table 2), although a high 5caC score was borderline significantly associated with BCR when analyzed as a dichotomized variable in univariate Cox regression (*p* = 0.058; Table 2) and Kaplan-Meier analyses (*p* = 0.055; log-rank test; Fig. 9b). In contrast, in *ERG*+ PCs, a low 5caC score (continuous and dichotomized) was significantly associated with early BCR in univariate Cox regression (*p* = 0.011/*p* = 0.034, Table 2) as well as in Kaplan-Meier analysis (*p* = 0.032; log-rank test; Fig. 9c). However, 5caC did not remain significant in multivariate Cox regression analysis after adjustment for routine clinicopathological parameters (5caC continuous/dichotomized: *p* = 0.299/*p* = 0.182; Additional file 17: Table S8/Additional file 18: Table S9).

In summary, high levels of 5hmC were significantly associated with shorter time to BCR in *ERG−* PCs, consistent with our previous report [30]. Additionally, in the present study, we found that high 5caC levels were significantly associated with favorable prognosis after RP in *ERG+* PCs, but were borderline significantly associated with poor prognosis in *ERG−* PCs. Finally, 5mC and 5fC did not show significant prognostic value in our RP cohort.

### Prognostic potential in ERG− PC for a combined 5hmC/5caC score

To investigate whether a 5hmC/5caC dual-marker panel could improve prognostic performance in *ERG−* PCs, three patient subgroups were defined: low (5hmC ≤ 1 and 5caC ≤ 1), moderate (5hmC ≤ 1 and 5caC > 1, or 5hmC > 1 and 5caC ≤ 1), and high 5hmC/5caC score (5hmC > 1 and 5caC > 1).

**Fig. 10:**
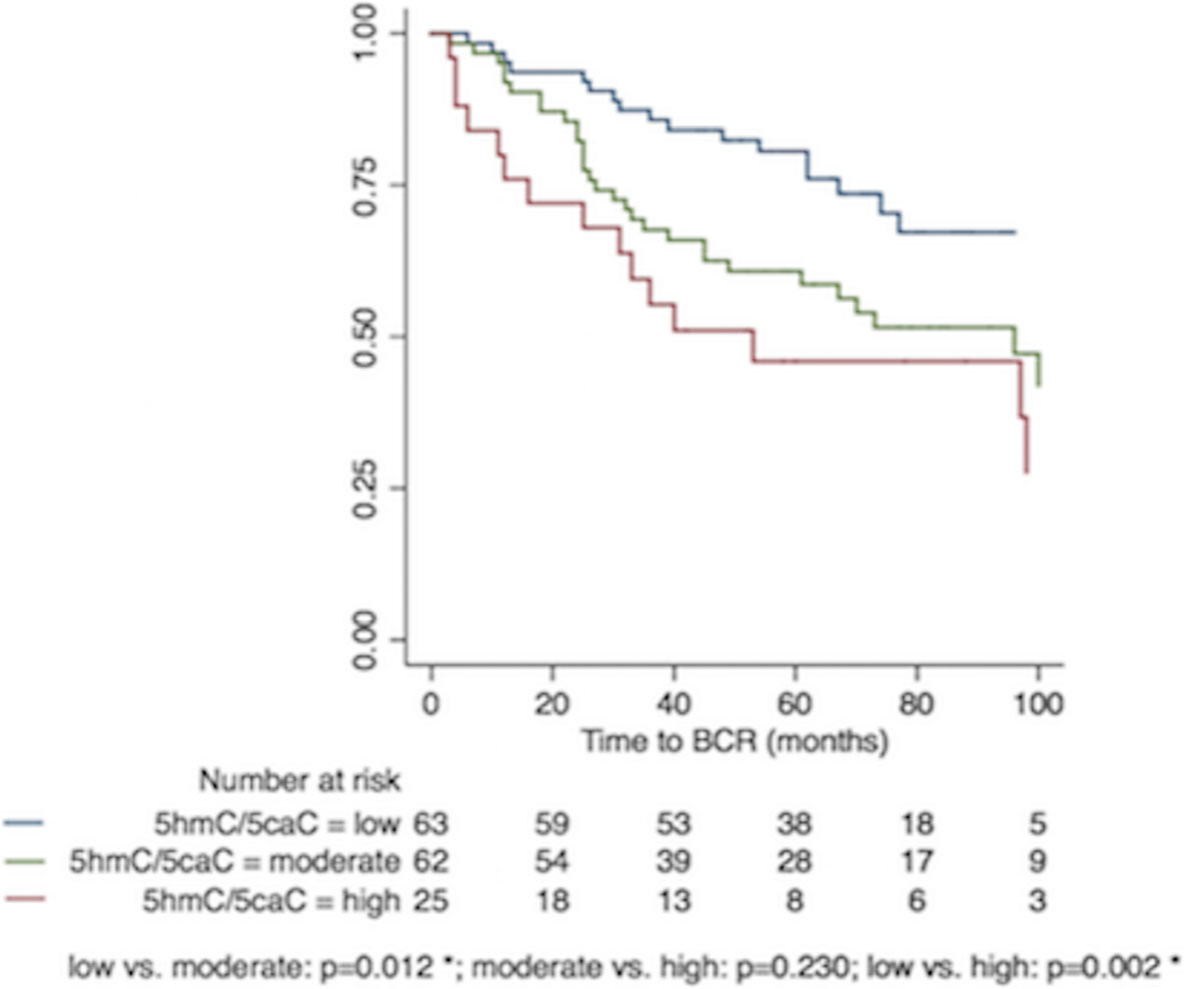
Kaplan-Meier analysis: Association between combined 5hmC/5caC score and time to BCR after RP in *ERG−* PC. To evaluate the prognostic potential of combined 5hmC/5caC IHC score in *ERG−* PCs, three patient subgroups were defined as low (5hmC ≤ 1 and 5caC ≤ 1; blue curve), moderate (5hmC ≤ 1 and 5caC > 1, or 5hmC > 1 and 5caC ≤ 1; green curve), and high (5hmC > 1 and 5caC > 1; red curve). The number of patients in each subgroup and *p* values for 2-sided log-rank tests are listed at the bottom. Significant *p* values are marked by an asterisk (*)

In *ERG−* PCs, a high 5hmC/5caC score was significantly associated with poor BCR-free survival in Kaplan-Meier analysis (low vs. high: *p* = 0.002; log-rank test; Fig. 10) and in univariate Cox regression analysis (low vs. high: hazard ration (HR) (95% confidence interval (CI)): 2.99 (1.49–6.02); *p* = 0.002; Table 3). Moreover, a high 5hmC/5caC score remained significant also after adjustment for routine clinical variables in multivariate Cox regression analysis (low vs. high: HR (95%CI): 2.48 (1.20–5.13); *p* = 0.014; Table 3). We used Harrell’s C-index to estimate predictive accuracy. In the final model, Harrell’s C-index improved from 0.69 to 0.75 when adding 5hmC/5caC score to a multivariate model based only on clinicopathological factors (Table 3). Similar analyses in the full PC patient set and in the *ERG+* PC subgroup, respectively, showed no significant associations between 5hmC/5caC score and time to BCR (data not shown). To the best of our knowledge, this is the first report to demonstrate a significant association between 5caC levels and PC outcome.

**Table 3:** Prognostic value of combined 5hmC/5caC IHC score in *ERG−* PC

*ERG*− PC patient subset (*n* = 150, 64 BCR)									
Variable	Univariate			Multivariate^a^		Multivariate^b^			
	HR (95% CI)	*p* value	C-index	HR (95% CI)	*p* value	HR (95% CI)	*p* value	C-index^c^	C-index^d^
5hmC/5caC (low vs. moderate)	2.06 (1.14–3.72)	*0.017*	0.62	1.79 (0.98–3.28)	0.057	–	–	0.75	–
5hmC/5caC (low vs. high)	2.99 (1.49–6.02)	*0.002*	0.62	2.48 (1.20–5.13)	*0.014*	3.18 (1.54–6.56)	*0.002*		–
Pre-op. PSA (≤ 10 vs. > 10 ng/ml)	2.65 (1.47–4.76)	*0.001*	0.60	2.79 (1.52–5.12)	*0.001*	4.22 (1.67–10.65)	*0.002*		0.69
Gleason score (< 7 vs. ≥ 7)	2.18 (1.27–3.72)	*0.005*	0.59	1.61 (0.88–2.94)	0.123	–	–		
Surgical margin (neg. vs. pos.)	2.63 (1.63–4.22)	*< 0.001*	0.61	2.04 (1.17–3.56)	*0.012*	4.17 (2.02–8.63)	*< 0.001*		
Tumor stage (≤pT2c vs. ≥pT3a)	2.55 (1.59–4.11)	*< 0.001*	0.61	1.70 (0.98–2.95)	0.060	**–**	–		

Univariate and multivariate Cox regression analyses of time to BCR using a combined 5hmC/5caC IHC score (low: 5hmC ≤ 1 and 5caC ≤ 1; moderate: 5hmC ≤ 1 and 5caC > 1, or 5hmC > 1 and 5caC ≤ 1; high: 5hmC > 1 and 5caC > 1)

Significant *p* values are highlighted in italics

^a^Global multivariate model including all parameters

^b^Final multivariate model including only significant variables

^c^Harrell’s C-index for final model including 5hmC/5caC

^d^Harrell’s C-index for final model excluding 5hmC/5caC

## Discussion

The present study is the first comprehensive investigation of 5mC, 5hmC, 5fC, and 5caC levels in PC. Based on IHC analysis of serial sections of a large tissue microarray, including NM and PC tissue samples from more than 500 RP patients, we observed significantly reduced levels of 5mC and 5hmC particularly in *ERG−* PCs. Furthermore, we found that 5fC levels were significantly increased in *ERG+* PCs, whereas 5caC levels were significantly elevated in both *ERG+* and *ERG−* PCs, as compared to NM prostate tissue samples. In addition, we observed significant positive correlations between the global levels of 5mC, 5fC, and 5caC in both NM and PC tissue samples, whereas 5hmC levels were weakly positively correlated only to 5mC levels and only in the PC subset. Moreover, high 5hmC levels were associated with poor BCR-free survival in *ERG−* PCs, consistent with our earlier findings for a smaller subset of patients in this RP cohort [30]. While there were no significant associations between 5mC, 5fC, or *ERG* status and BCR-free survival in our RP cohort, we found that high 5caC levels were significantly associated with favorable prognosis in *ERG+* PCs, while at the same time being borderline significantly associated with poor prognosis in *ERG−* PCs. Moreover, in *ERG−* PCs, a combined high-5hmC/high-5caC score was a significant adverse predictor of post-operative BCR beyond routine clinicopathological variables.

Our current findings for 5mC and 5hmC immunoreactivity patterns in PC compared to NM prostate tissues confirm and expand on previous reports of global DNA methylation loss in PC as well as in other malignancies [38]. Consistent with our results, two previous small-scale studies observed reduced 5mC immunoreactivity in PC tissue samples based on analysis of 48 NM vs. 48 PC [39] and 10 NM vs. 14 PC samples [20], respectively. Similarly, two earlier small-scale studies reported that 5hmC levels were reduced in PC tissue samples based on IHC analyses of 10 NM vs. 30 PC [20] and 11 NM vs. 11 PC [14] samples, respectively. However, as opposed to our present study, none of these earlier studies [14, 20, 39] distinguished clearly between 5mC and 5hmC, while also stratifying for *ERG* fusion status. We have recently reported that 5hmC immunoreactivity levels were reduced particularly in *ERG−* PCs, based on analysis of smaller subset of patients from this RP cohort [30]. Here, we confirmed these results and extended our analyses to three additional DNA methylation marks (5mC, 5fC and 5caC), while also stratifying for *ERG* status.

The present study is the first to describe 5fC and 5caC immunoreactivity patterns in NM and PC tissue samples. We found that 5fC levels were significantly elevated in *ERG+* PCs, while 5caC levels were significantly increased in both *ERG+* and *ERG−* PCs. It has previously been reported that global 5caC levels are increased in breast cancer and glioma compared to their corresponding normal tissues [21], together indicating that global 5caC alterations are associated with malignant transformation in multiple cancer types. However, future studies are needed to investigate this in more detail. Furthermore, to fully understand the epigenetic reprogramming mechanisms associated with PC development and progression, such future studies should include not only an assessment of the global levels of 5caC and 5fC in NM and PCs (*ERG+* vs. *ERG−*), but should also map the genome-wide distribution of these marks as compared to 5mC and 5hmC, ideally at single-base resolution. Moreover, since reactive oxygen species and hypoxia can induce TET expression [40–42], it cannot be excluded that variable levels of tumor hypoxia may have affected the levels of 5hmC, 5fC, and/or 5caC observed in our study. Thus, further studies are needed to investigate the possible associations between hypoxia and TET-dependent epigenetic marks in PC, but is considered beyond the scope of the present work.

We are the first to demonstrate significant positive correlations between matching 5mC, 5fC, and 5caC global levels in both PC and NM samples. For 5hmC, we observed only a weak positive correlation with 5mC, which is consistent with several previous reports, suggesting that 5hmC is an independent epigenetic mark, while 5fC and 5caC are more likely to be short-lived intermediates in the active demethylation processes [17, 20, 34, 35]. Yet, our correlation analysis results are also consistent with the possibility that 5fC and/or 5caC hold independent regulatory roles, as suggested by the identification of proteins that bind specifically to 5fC or 5caC [18, 19, 43]. It has also been reported that 5fC can be stably detected in vivo, favoring a possible biological role for 5fC beyond that of a demethylation intermediate [44]. Likewise, the significant prognostic value demonstrated for 5caC in the present study might be interpreted in favor of a possible independent regulatory role for this mark. Further studies are needed to investigate this, but are beyond the scope of the current work.

We assessed the prognostic potential of 5mC, 5hmC, 5fC, and 5caC in a large RP cohort using BCR-free survival as the clinical endpoint. There was no significant association between 5mC immunoreactivity and BCR in this RP cohort, also not after stratification for *ERG* status. This is in accordance with results from an earlier small-scale study that also found no prognostic value for 5mC immunoreactivity in PC (*n* = 48) [39]. In contrast, global loss of 5mC has been associated with poor prognosis in tongue squamous cell carcinoma [45], while increased 5mC levels in myelodysplastic syndrome have been linked with a worse prognosis [46].

Consistent with our previous results for a smaller subset of patients in this RP cohort [30], we found that high 5hmC levels were significantly associated with shorter BCR-free survival in *ERG−* PCs. Likewise, high levels of 5hmC have previously been associated with poor prognosis in AML [29]. Conversely, in several other cancers, including esophageal squamous cell carcinoma [47], diffuse astrocytoma [22], NSCLC [24], cervical squamous cell carcinoma [25], gastric cancer [28], and malignant melanoma [48], poor prognosis has been associated with low 5hmC immunoreactivity. Disease-specific differences may likely reflect that phenotypic effects of epigenetic deregulation are influenced not only by the global level of specific DNA methylation marks, but also by their genomic distribution in any given cell type.

The potential prognostic value of 5fC or 5caC has not previously been evaluated in relation to cancer in general or to PC in particular. Here, we found no significant associations between 5fC immunoreactivity and BCR-free survival in our RP cohort. In contrast, high 5caC levels were significantly associated with favorable outcome in *ERG+* PCs, while at the same time being borderline significantly associated with poor prognosis in *ERG−* PCs. In addition, we found that the combination of high 5hmC and high 5caC score in *ERG−* PCs were significantly associated with shorter BCR-free survival and thereby considerably worse prognosis (HR > 3) after adjustment for routine clinicopathological factors. Although further validation is needed, this suggests that a 5hmC/5caC dual-marker panel has the potential to help improve risk stratification for this patient subset. Better and more accurate risk stratification is crucial for PC patient management, as it could be used to guide more individualized treatment decisions in the future.

There are some limitations to the present study. First, our analyses were restricted to patients who underwent RP for clinically localized PC. Accordingly, conclusions cannot necessarily be transferred to PC patients with advanced/metastatic disease. Nevertheless, it is considered to be a strength of the current study that our results are based on a large consecutive and representative RP cohort from one clinical center with clinical annotation and follow-up information available for all patients. Furthermore, our study was based on IHC staining, which only allows assessment of global 5mC, 5hmC, 5fC, and 5caC levels. Thus, future studies are needed to map the genome-wide distribution of these marks at single-base resolution in NM and PC tissue samples. We did not apply multiple testing correction to the statistical analyses, as each methylation mark was analyzed individually. However, the main results (prognostic value of 5hmC score in *ERG−* PC and of 5caC score in *ERG+* PC; Table 2) would also have remained significant after correction for multiple testing, even if using the most stringent Bonferroni correction method.

Another possible limitation is the use of BCR as endpoint for prognostic biomarker evaluation, as BCR is known to be only a surrogate for PC aggressiveness. However, due to the slow-growing nature of PC, we did not have sufficient numbers of events for metastatic progression and/or PC-specific mortality analyses. Moreover, as we did not have access to primary and secondary Gleason grades from all patients, our prognostic analyses did not distinguish Gleason 3 + 4 vs. 4 + 3, although these are generally accepted as separate risk groups in the clinic. Finally, our study was restricted to one large RP cohort from Denmark and further independent validation is needed. Future validation studies should include multiple large PC patient cohorts with full clinical annotation, long clinical follow-up, and representing different ethnic populations.

## Conclusions

To the best of our knowledge, this is the first study to analyze 5fC and 5caC immunoreactivity patterns in NM and PC tissue samples as well as the first report to demonstrate a significant association between 5caC levels and PC outcome. The results from our parallel IHC analyses of 5mC, 5hmC, 5fC, and 5caC in NM and PC tissue samples from more than 500 RP patients support the notion that epigenetic deregulation is a molecular hallmark of PC, and furthermore suggest that PC-associated epigenetic reprogramming differs between *ERG+* and *ERG−* PCs. Future studies are warranted to further investigate this.

### Availability of supporting data

The datasets supporting the conclusions of this article are included within the article and its additional files.

## Additional files

Additional file 1: **Figure S1.** Flow chart illustrating the sample inclusion/exclusion process in malignant cores. A) 5mC score. B) 5hmC score. **C)** 5fC score. D) 5caC score. N, number of patients. (PNG 931 kb)

Additional file 2: **Figure S2.** Flow chart illustrating the sample inclusion/exclusion process in NM cores. A) 5mC score. B) 5hmC score. C) 5fC score. D) 5caC score. N, number of patients. (PNG 1027 kb)

Additional file 3: **Figure S3.** Correlations between 5mC score and clinicopathological parameters. A) In the full PC set (*n* = 344), B) in *ERG−* PC (*n* = 150), and C) in *ERG+* PC (*n* = 192). Significant *p* values (chi^2^ test) are marked by an asterisk (*). (JPG 392 kb)

Additional file 4: **Figure S4.** Correlations between 5hmC score and clinicopathological parameters. A) In the full PC set (*n* = 367), B) in *ERG−* PCs (*n* = 161), and C) in *ERG+* PCs (*n* = 206). Significant *p* values (chi^2^ test) are marked by an asterisk (*). (JPG 406 kb)

Additional file 5: **Figure S5.** Correlations between 5fC score and clinicopathological parameters. A) In the full PC set (*n* = 281), B) in *ERG−* PC (*n* = 135), and C) in *ERG+* PC (*n* = 138). Significant *p* values (chi^2^ test) are marked by an asterisk (*). (JPG 407 kb)

Additional file 6: **Figure S6.** Correlations between 5caC score and clinicopathological parameters. A) In the full PC set (*n* = 351), B) in *ERG−* PC (*n* = 160), and C) in *ERG+* PC (*n* = 187). Significant *p* values (chi^2^ test) are marked by an asterisk (*). (JPG 415 kb)

Additional file 7: **Table S1A.** Clinical characteristics for PC patients represented on the TMA. Data for RP patients for whom a 5mC score could be evaluated in malignant cores. Two PC specimens had unknown *ERG* status. (DOCX 15 kb)

Additional file 8: **Table S1B.** Clinical characteristics for PC patients represented on the TMA. Data for RP patients for whom a 5hmC score could be evaluated in malignant cores. (DOCX 15 kb)

Additional file 9: **Table S1C.** Clinical characteristics for PC patients represented on the TMA. Data for RP patients for whom a 5fC score could be evaluated in malignant cores. Eight PC specimens had unknown *ERG* status. (DOCX 15 kb)

Additional file 10: **Table S1D.** Clinical characteristics for PC patients represented on the TMA. Data for RP patients for whom a 5caC score could be evaluated in malignant cores. Four PC specimens had unknown *ERG* status. (DOCX 15 kb)

Additional file 11: **Table S2.** Details for the antibodies used for IHC (DOCX 14 kb)

Additional file 12: **Table S3.** Correlation between the methylation marks in PC and NM specimens. Correlation between the methylation marks in PC (*n* = 232) and NM (*n* = 209) specimens evaluated with Spearman’s rank correlation coefficient (rho, *ρ*) based on mean IHC scores. Significant *p* values are highlighted in bold. (DOCX 14 kb)

Additional file 13: **Table S4.** 5mC score (continuous and dichotomized) in univariate Cox regression analysis of BCR-free survival. Significant *p* values are highlighted in bold. (DOCX 16 kb)

Additional file 14: **Table S5.** 5hmC score (continuous) in univariate and multivariate Cox regression analysis of BCR-free survival. (DOCX 18 kb)

Additional file 15: **Table S6.** 5hmC score (dichotomized) in univariate and multivariate Cox regression analysis of BCR-free survival. (DOCX 19 kb)

Additional file 16: **Table S7.** 5fC score (continuous and dichotomized) in univariate Cox regression analysis of BCR-free survival. (DOCX 15 kb)

Additional file 17: **Table S8.** 5caC score (continuous) in univariate and multivariate Cox regression analysis of BCR-free survival. (DOCX 16 kb)

Additional file 18: **Table S9.** 5caC score (dichotomized) in univariate and multivariate Cox regression analysis of BCR-free survival. (DOCX 20 kb)

## Abbreviations

5caC: 5-carboxylcytosine
5fC: 5-formylcytosine
5hmC: 5-hydroxymethylcytosine
5mC: 5-methylcytosine
BCR: Biochemical recurrence
CI: Confidence interval
DAB: Diaminobenzidine
DNMTs: DNA methyltransferases
*ERG*: ETS-related gene
*ERG+*: *ERG* positive
*ERG−*: *ERG* negative
GS: Gleason score
HR: Hazard ratio
HRP: Horse radish peroxidase
IHC: Immunohistochemistry
NM: Adjacent non-malignant
PC: Prostate cancer
PSA: Prostate-specific antigen
pT stage: Pathological tumor stage
RP: Radical prostatectomy
SM: Surgical margin
*TET*: Ten-eleven translocation
TMA: Tissue microarray
*TMPRSS2*: Transmembrane protease, serine 2
