# Supplementary figures and images for: Dysregulation and prognostic potential of 5-methylcytosine (5mC), 5-hydroxymethylcytosine (5hmC), 5-formylcytosine (5fC), and 5-carboxylcytosine (5caC) levels in prostate cancer
Source: Clin Epigenetics. 2018 Aug 7;10:105. doi: 10.1186/s13148-018-0540-x (PMC6081903; doi:10.1186/s13148-018-0540-x)

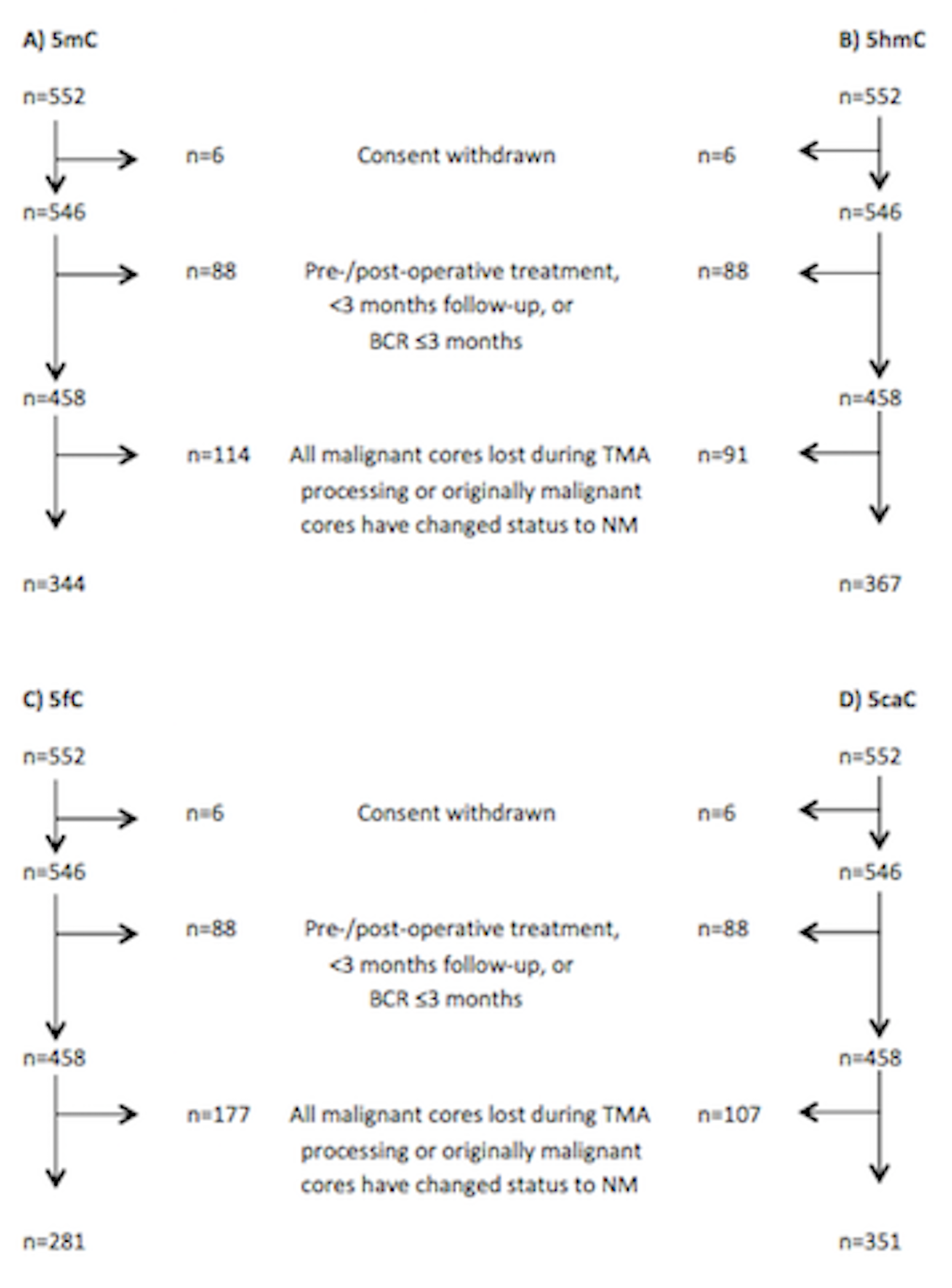

Supplement: Supplementary file 1 — Figure S1. Flow chart illustrating the sample inclusion/exclusion process in malignant cores. A) 5mC score. B) 5hmC score. C) 5fC score. D) 5caC score. N, number of patients. (PNG 931 kb) [file 13148_2018_540_MOESM1_ESM.png]

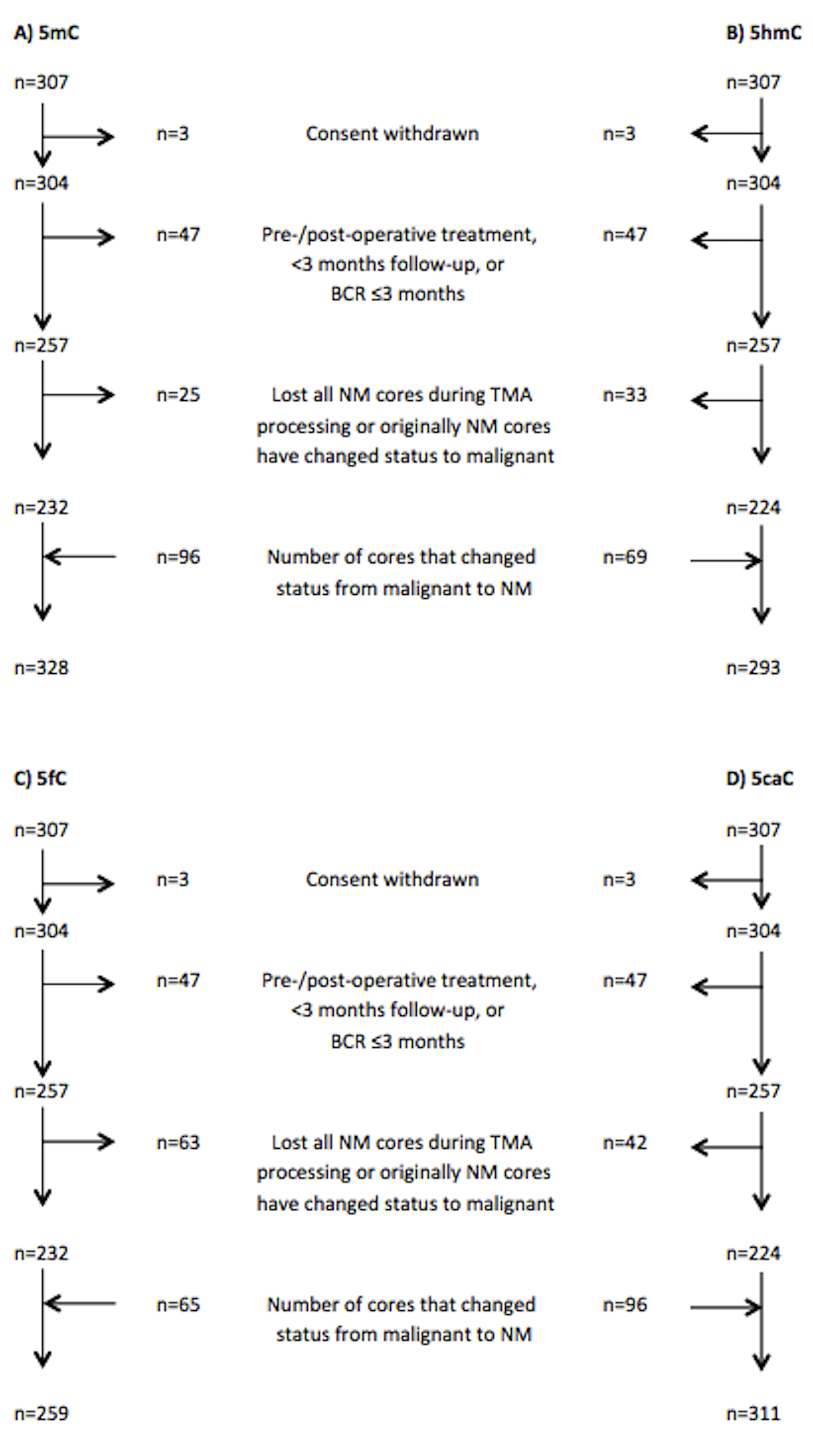

Supplement: Supplementary file 2 — Figure S2. Flow chart illustrating the sample inclusion/exclusion process in NM cores. A) 5mC score. B) 5hmC score. C) 5fC score. D) 5caC score. N, number of patients. (PNG 1027 kb) [file 13148_2018_540_MOESM2_ESM.png]

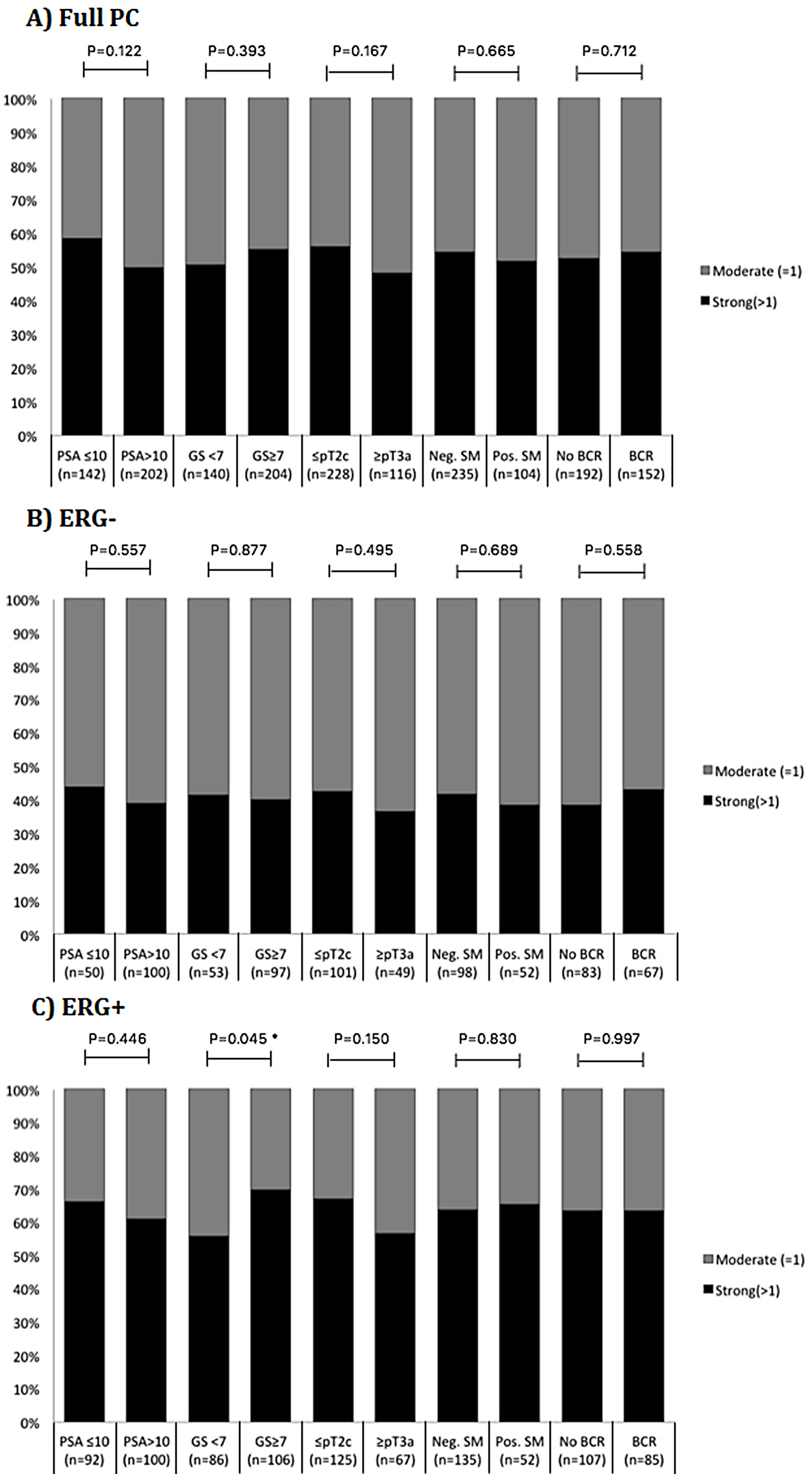

Supplement: Supplementary file 3 — Figure S3. Correlations between 5mC score and clinicopathological parameters. A) In the full PC set (n = 344), B) in ERG− PC (n = 150), and C) in ERG+ PC (n = 192). Significant p values (chi2 test) are marked by an asterisk (*). (JPG 392 kb) [file 13148_2018_540_MOESM3_ESM.jpg]

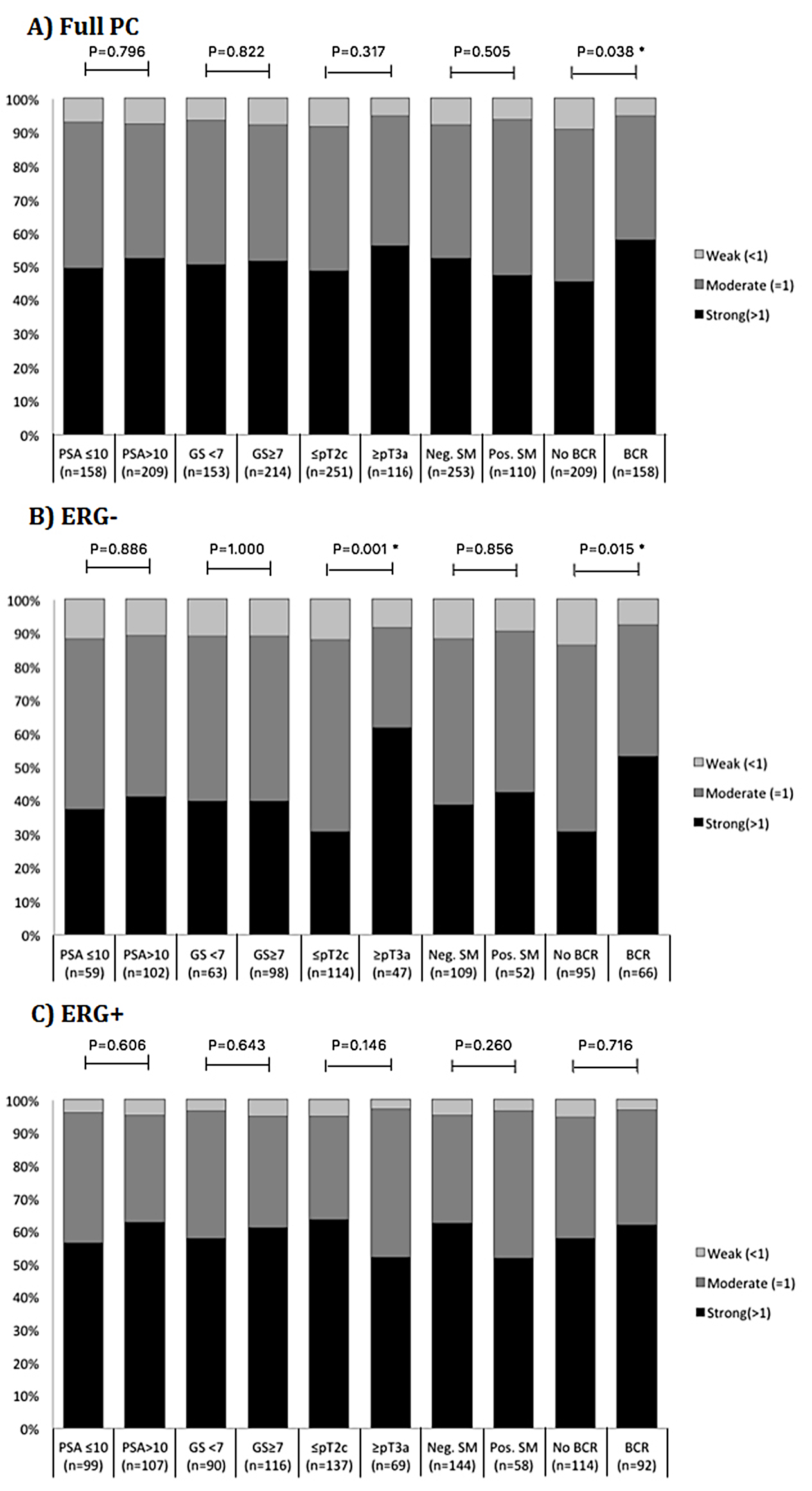

Supplement: Supplementary file 4 — Figure S4. Correlations between 5hmC score and clinicopathological parameters. A) In the full PC set (n = 367), B) in ERG− PCs (n = 161), and C) in ERG+ PCs (n = 206). Significant p values (chi2 test) are marked by an asterisk (*). (JPG 406 kb) [file 13148_2018_540_MOESM4_ESM.jpg]

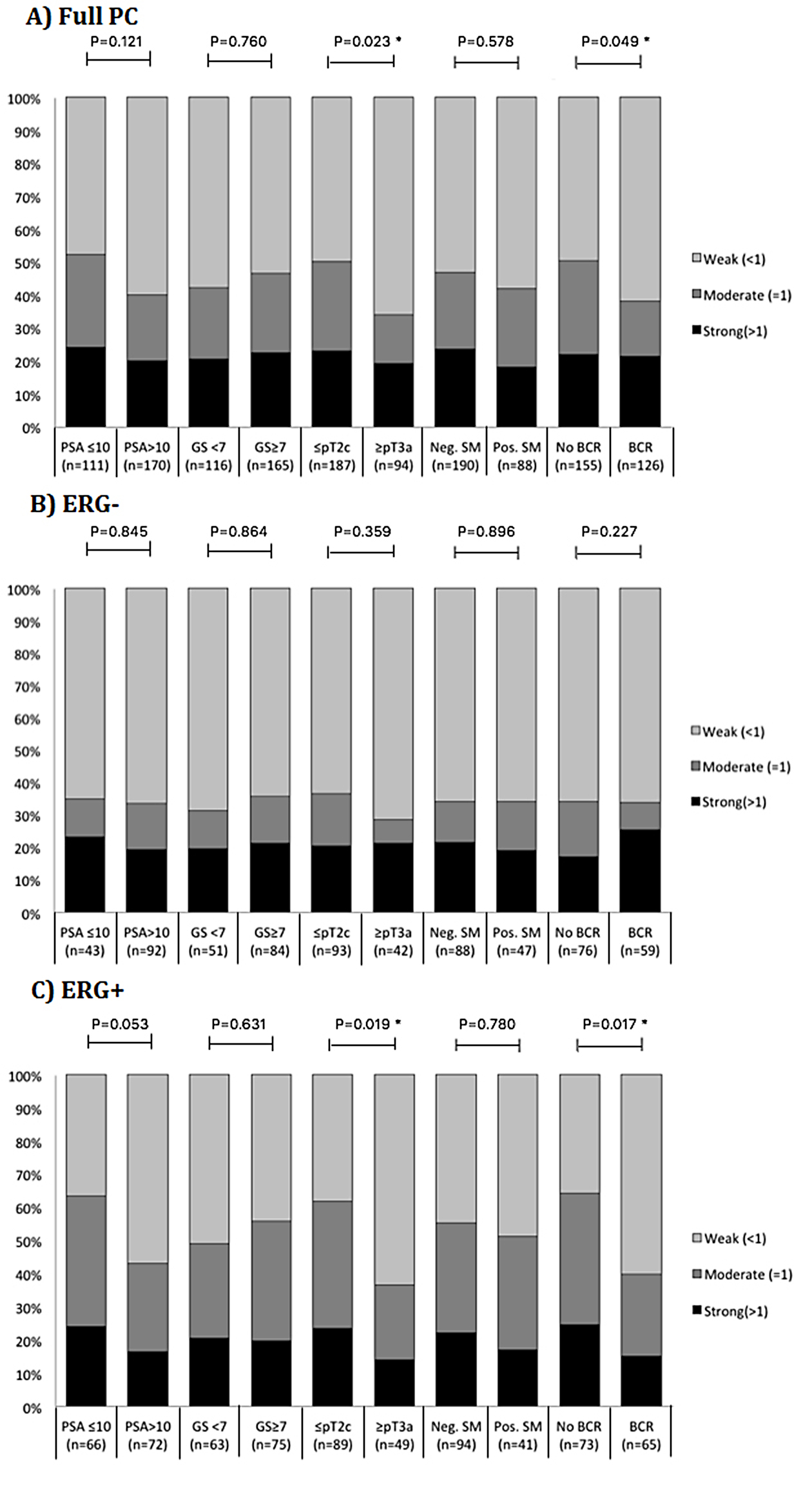

Supplement: Supplementary file 5 — Figure S5. Correlations between 5fC score and clinicopathological parameters. A) In the full PC set (n = 281), B) in ERG− PC (n = 135), and C) in ERG+ PC (n = 138). Significant p values (chi2 test) are marked by an asterisk (*). (JPG 407 kb) [file 13148_2018_540_MOESM5_ESM.jpg]

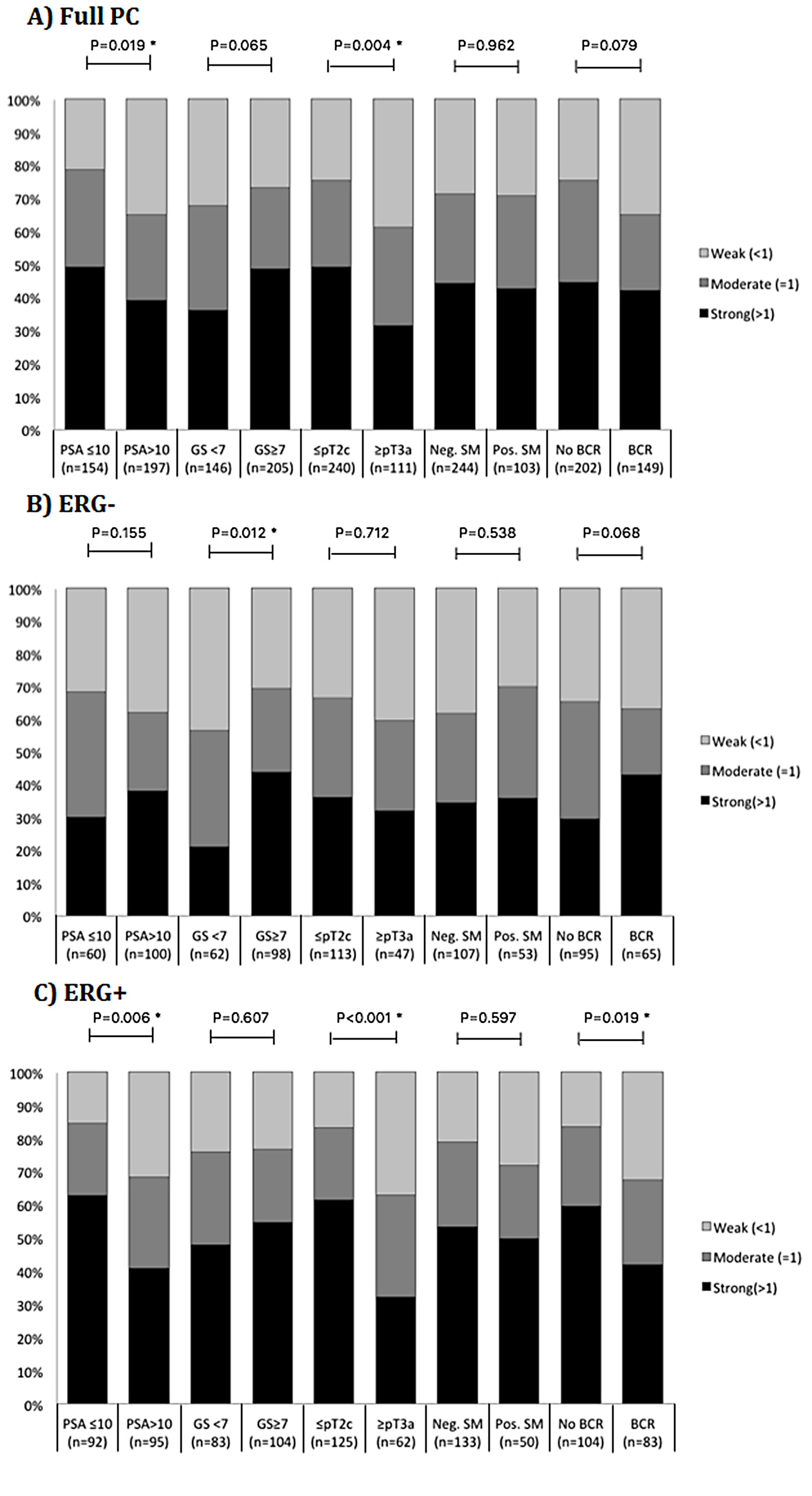

Supplement: Supplementary file 6 — Figure S6. Correlations between 5caC score and clinicopathological parameters. A) In the full PC set (n = 351), B) in ERG− PC (n = 160), and C) in ERG+ PC (n = 187). Significant p values (chi2 test) are marked by an asterisk (*). (JPG 415 kb) [file 13148_2018_540_MOESM6_ESM.jpg]
