# Supplementary material for: Dysregulation and prognostic potential of 5-methylcytosine (5mC), 5-hydroxymethylcytosine (5hmC), 5-formylcytosine (5fC), and 5-carboxylcytosine (5caC) levels in prostate cancer
Source: Clin Epigenetics. 2018 Aug 7;10:105. doi: 10.1186/s13148-018-0540-x (PMC6081903; doi:10.1186/s13148-018-0540-x)
Supplement: Supplementary file 7 — Table S1A. Clinical characteristics for PC patients represented on the TMA. Data for RP patients for whom a 5mC score could be evaluated in malignant cores. Two PC specimens had unknown ERG status. (DOCX 15 kb) [file 13148_2018_540_MOESM7_ESM.docx]

**Additional file 7: Table S1A.**

**Clinical characteristics for PC patients represented on the TMA**

| **5-methylcytosine** | **546 RP patients included on TMA** | **RP malignant cores**  **n=344** | ***ERG*-**  **n= 150** | ***ERG+***  **n= 192** |
| --- | --- | --- | --- | --- |
| **Age at RP (years), median (range)** | 63 (34-76) | 63 (34-76) | 64 (34-76) | 62 (47-74) |
| **Pathological GS** |  |  |  |  |
| <7, n (%) | 229 (41.9) | 140 (40.7) | 53 (35.3) | 86 (44.8) |
| ≥7, n (%) | 317 (58.1) | 204 (59.3) | 97 (64.7) | 106 (55.2) |
| **Pathological T stage** |  |  |  |  |
| ≤ pT2c, n (%) | 363 (66.5) | 228 (66.3) | 101 (67.3) | 125 (65.1) |
| ≥ pT3a, n (%) | 182 (33.3) | 116 (33.7) | 49 (32.7) | 67 (34.9) |
| Unknown | 1 (0.2) | - | - | - |
| **Preoperative PSA** |  |  |  |  |
| PSA ≤ 10 ng/ml, n (%) | 222 (40.7) | 142 (41.3) | 50 (33.3) | 92 (47.9) |
| PSA >10 ng/ml, n (%) | 324 (59.3) | 202 (58.7) | 100 (66.7) | 100 (52.1) |
| **Surgical margin status** |  |  |  |  |
| Negative, n (%) | 366 (67.0) | 235 (68.3) | 98 (65.3) | 135 (70.3) |
| Positive, n (%) | 175 (32.1) | 104 (30.2) | 52 (34.7) | 52 (27.1) |
| Unknown, n (%) | 5 (0.9) | 5 (1.5) | - | 5 (2.6) |
| **Follow-up (months), median (range)** | 80 (12-158) | 81.5 (12-152) | 81 (23-148) | 82 (12-152) |
| **BCR** |  |  |  |  |
| No, n (%) | 310 (56.8) | 192 (55.8) | 83 (55.3) | 107 (55.7) |
| Yes, n (%) | 236 (43.2) | 152 (44.2) | 67 (44.7) | 85 (44.3) |

Data for RP patients for whom a 5mC score could be evaluated in malignant cores. Two PC specimens had unknown ERG status.
