# Supplementary material for: Dysregulation and prognostic potential of 5-methylcytosine (5mC), 5-hydroxymethylcytosine (5hmC), 5-formylcytosine (5fC), and 5-carboxylcytosine (5caC) levels in prostate cancer
Source: Clin Epigenetics. 2018 Aug 7;10:105. doi: 10.1186/s13148-018-0540-x (PMC6081903; doi:10.1186/s13148-018-0540-x)
Supplement: Supplementary file 8 — Table S1B. Clinical characteristics for PC patients represented on the TMA. Data for RP patients for whom a 5hmC score could be evaluated in malignant cores. (DOCX 15 kb) [file 13148_2018_540_MOESM8_ESM.docx]

**Additional file 8: Table S1B.**

**Clinical characteristics for PC patients represented on the TMA**

| **5-hydroxymethylcytosine** | **546 RP patients**  **included on**  **TMA** | **RP malignant cores**  **n=367** | ***ERG-***  **n= 161** | ***ERG+***  **n= 206** |
| --- | --- | --- | --- | --- |
| **Age at RP (years), median (range)** | 63 (34-76) | 63 (34-76) | 64 (34-76) | 62.5 (48-74) |
| **Pathological GS** |  |  |  |  |
| <7, n (%) | 229 (41.9) | 153 (41.7) | 63 (39.1) | 90 (43.7) |
| ≥7, n (%) | 317 (58.1) | 214 (58.3) | 98 (60.9) | 116 (56.3) |
| **Pathological T stage** |  |  |  |  |
| ≤ pT2c, n (%) | 363 (66.5) | 251 (68.4) | 114 (70.8) | 137 (66.5) |
| ≥ pT3a, n (%) | 182 (33.3) | 116 (31.6) | 47 (29.2) | 69 (33.5) |
| Unknown | 1 (0.2) | - | - | - |
| **Preoperative PSA** |  |  |  |  |
| PSA ≤ 10 ng/ml, n (%) | 222 (40.7) | 158 (43.1) | 59 (36.7) | 99 (48.1) |
| PSA >10 ng/ml, n (%) | 324 (59.3) | 209 (56.9) | 102 (63.3) | 107 (51.9) |
| **Surgical margin status** |  |  |  |  |
| Negative, n (%) | 366 (67.0) | 253 (68.9) | 109 (67.7) | 144 (69.9) |
| Positive, n (%) | 175 (32.1) | 110 (30.0) | 52 (32.3) | 58 (28.2) |
| Unknown, n (%) | 5 (0.9) | 4 (1.1) | - | 4 (1.9) |
| **Follow-up (months), median (range)** | 80 (12-158) | 80 (12-158) | 80 (14-148) | 81 (12-158) |
| **BCR** |  |  |  |  |
| No, n (%) | 310 (56.8) | 209 (56.9) | 95 (59.0) | 114 (55.3) |
| Yes, n (%) | 236 (43.2) | 158 (43.1) | 66 (41.0) | 92 (44.7) |

Data for RP patients for whom a 5hmC score could be evaluated in malignant cores.
