# Supplementary material for: Dysregulation and prognostic potential of 5-methylcytosine (5mC), 5-hydroxymethylcytosine (5hmC), 5-formylcytosine (5fC), and 5-carboxylcytosine (5caC) levels in prostate cancer
Source: Clin Epigenetics. 2018 Aug 7;10:105. doi: 10.1186/s13148-018-0540-x (PMC6081903; doi:10.1186/s13148-018-0540-x)
Supplement: Supplementary file 9 — Table S1C. Clinical characteristics for PC patients represented on the TMA. Data for RP patients for whom a 5fC score could be evaluated in malignant cores. Eight PC specimens had unknown ERG status. (DOCX 15 kb) [file 13148_2018_540_MOESM9_ESM.docx]

Clinical characteristics for PC patients represented on the TMA

| **5-formylcytosine** | **546 RP patients included on TMA** | **RP malignant cores**  **n=281** | ***ERG-***  **n= 135** | ***ERG+***  **n= 138** |
| --- | --- | --- | --- | --- |
| **Age at RP (years), median (range)** | 63 (34-76) | 63 (48-76) | 64 (48-76) | 62 (49-72) |
| **Pathological GS** |  |  |  |  |
| <7, n (%) | 229 (41.9) | 116 (41.3) | 51 (37.8) | 63 (45.7) |
| ≥7, n (%) | 317 (58.1) | 165 (58.7) | 84 (62.2) | 75 (54.3) |
| **Pathological T stage** |  |  |  |  |
| ≤ pT2c, n (%) | 363 (66.5) | 187 (66.5) | 93 (68.9) | 89 (64.5) |
| ≥ pT3a, n (%) | 182 (33.3) | 94 (33.5) | 42 (31.1) | 49 (35.5) |
| Unknown | 1 (0.2) | - | - | - |
| **Preoperative PSA** |  |  |  |  |
| PSA ≤ 10 ng/ml, n (%) | 222 (40.7) | 111 (39.5) | 43 (31.9) | 66 (47.8) |
| PSA >10 ng/ml, n (%) | 324 (59.3) | 170 (60.5) | 92 (68.1) | 72 (52.2) |
| **Surgical margin status** |  |  |  |  |
| Negative, n (%) | 366 (67.0) | 190 (67.6) | 88 (65.2) | 94 (68.1) |
| Positive, n (%) | 175 (32.1) | 88 (31.3) | 47 (34.8) | 41 (29.7) |
| Unknown, n (%) | 5 (0.9) | 3 (1.1) | - | 3 (2.2) |
| **Follow-up (months), median (range)** | 80 (12-158) | 80 (12-158) | 79 (23-148) | 83 (12-158) |
| **BCR** |  |  |  |  |
| No, n (%) | 310 (56.8) | 155 (55.2) | 76 (56.3) | 73 (52.9) |
| Yes, n (%) | 236 (43.2) | 126 (44.8) | 59 (43.7) | 65 (47.1) |

Data for RP patients for whom a 5fC score could be evaluated in malignant cores. Eight PC specimens had unknown *ERG* status
