# Supplementary material for: Dysregulation and prognostic potential of 5-methylcytosine (5mC), 5-hydroxymethylcytosine (5hmC), 5-formylcytosine (5fC), and 5-carboxylcytosine (5caC) levels in prostate cancer
Source: Clin Epigenetics. 2018 Aug 7;10:105. doi: 10.1186/s13148-018-0540-x (PMC6081903; doi:10.1186/s13148-018-0540-x)
Supplement: Supplementary file 10 — Table S1D. Clinical characteristics for PC patients represented on the TMA. Data for RP patients for whom a 5caC score could be evaluated in malignant cores. Four PC specimens had unknown ERG status. (DOCX 15 kb) [file 13148_2018_540_MOESM10_ESM.docx]

**Additional file 10: Table S1D.**

**Clinical characteristics for PC patients represented on the TMA**

| **5-carboxylcytosine** | **546 RP patients included on TMA** | **RP malignant cores**  **n= 351** | ***ERG-***  **n= 160** | ***ERG+***  **n= 187** |
| --- | --- | --- | --- | --- |
| **Age at RP (years), median (range)** | 63 (34-76) | 63 (34-76) | 63 (34-76) | 62 (48-74) |
| **Pathological GS** |  |  |  |  |
| <7, n (%) | 229 (41.9) | 146 (41.6) | 62 (38.8) | 83 (44.4) |
| ≥7, n (%) | 317 (58.1) | 205 (58.4) | 98 (61.3) | 104 (55.6) |
| **Pathological T stage** |  |  |  |  |
| ≤ pT2c, n (%) | 363 (66.5) | 240 (68.4) | 113 (70.6) | 125 (66.8) |
| ≥ pT3a, n (%) | 182 (33.3) | 111 (31.6) | 47 (29.4) | 62 (33.2) |
| Unknown | 1 (0.2) | - | - | - |
| **Preoperative PSA** |  |  |  |  |
| PSA ≤ 10 ng/ml, n (%) | 222 (40.7) | 154 (43.9) | 60 (37.5) | 92 (49.2) |
| PSA >10 ng/ml, n (%) | 324 (59.3) | 197 (56.1) | 100 (62.5) | 95 (50.8) |
| **Surgical margin status** |  |  |  |  |
| Negative, n (%) | 366 (67.0) | 244 (69.5) | 107 (66.9) | 133 (71.1) |
| Positive, n (%) | 175 (32.1) | 103 (29.3) | 53 (33.1) | 50 (26.8) |
| Unknown, n (%) | 5 (0.9) | 4 (1.2) | - | 4 (2.1) |
| **Follow-up (months), median (range)** | 80 (12-158) | 81 (12-158) | 81 (19-148) | 82 (12-158) |
| **BCR** |  |  |  |  |
| No, n (%) | 310 (56.8) | 202 (57.6) | 95 (59.4) | 104 (55.6) |
| Yes, n (%) | 236 (43.2) | 149 (42.4) | 65 (40.6) | 83 (44.4) |

Data for RP patients for whom a 5caC score could be evaluated in malignant cores. Four PC specimens had unknown *ERG* status.
