# Supplementary material for: Dysregulation and prognostic potential of 5-methylcytosine (5mC), 5-hydroxymethylcytosine (5hmC), 5-formylcytosine (5fC), and 5-carboxylcytosine (5caC) levels in prostate cancer
Source: Clin Epigenetics. 2018 Aug 7;10:105. doi: 10.1186/s13148-018-0540-x (PMC6081903; doi:10.1186/s13148-018-0540-x)
Supplement: Supplementary file 11 — Table S2. Details for the antibodies used for IHC (DOCX 14 kb) [file 13148_2018_540_MOESM11_ESM.docx]

**Additional file 11: Table S2.**

**Details for the antibodies used for IHC**

| **Antibody** | **Company** | **Cat. No.** | **Dilution** | **Buffer** | **Origin** | **Clonality** |
| --- | --- | --- | --- | --- | --- | --- |
| **5mC** | Active Motif | 39649 | 1:250 | TEG pH 9.00 | Mouse | Monoclonal |
| **5hmC** | Active Motif | 39769 | 1:1000 | Citrate pH 6.00 | Rabbit | Polyclonal |
| **5fC** | Active Motif | 61223 | 1:500 | Citrate pH 6.00 | Rabbit | Polyclonal |
| **5caC** | Active Motif | 61225 | 1:1000 | Citrate pH 6.00 | Rabbit | Polyclonal |
| ***ERG*** | Epitomics | 2805-1 | 1:150 | TEG pH 9.00 | Rabbit | Monoclonal |
