# Supplementary material for: Dysregulation and prognostic potential of 5-methylcytosine (5mC), 5-hydroxymethylcytosine (5hmC), 5-formylcytosine (5fC), and 5-carboxylcytosine (5caC) levels in prostate cancer
Source: Clin Epigenetics. 2018 Aug 7;10:105. doi: 10.1186/s13148-018-0540-x (PMC6081903; doi:10.1186/s13148-018-0540-x)
Supplement: Supplementary file 12 — Table S3. Correlation between the methylation marks in PC and NM specimens. Correlation between the methylation marks in PC (n = 232) and NM (n = 209) specimens evaluated with Spearman’s rank correlation coefficient (rho, ρ) based on mean IHC scores. Significant p values are highlighted in bold. (DOCX 14 kb) [file 13148_2018_540_MOESM12_ESM.docx]

**Additional file 12: Table S3.**

**Correlation between the methylation marks in PC and NM specimens**

| **PC**  n=232 | **5mC** | | **5hmC** | **5fC** | **5caC** |
| --- | --- | --- | --- | --- | --- |
| **5mC** |  | |  |  |  |
| **5hmC** | *ρ=*0.143  p=**0.030** | |  |  |  |
| **5fC** | *ρ=*0.481  p**<0.001** | | *ρ*=0.119  p=0.072 |  |  |
| **5caC** | *ρ*=0.490  p**<0.001** | | *ρ*=0.041  p=0.538 | *ρ*=0.513  p**<0.001** |  |
| **NM**  n=209 | | **5mC** | **5hmC** | **5fC** | **5caC** |
| **5mC** | |  |  |  |  |
| **5hmC** | | *ρ=*0.098  p=0.159 |  |  |  |
| **5fC** | | *ρ=*0.529  p**<0.001** | *ρ*=0.007  p=0.917 |  |  |
| **5caC** | | *ρ*=0.430  p**<0.001** | *ρ*=0.026  p=0.712 | *ρ*=0.504  p**<0.001** |  |

Correlation between the methylation marks in PC (n=232) and NM (n=209) specimens evaluated with Spearman’s rank correlation coefficient (rho, ), based on mean IHC scores. Significant p-values are highlighted in bold.
