# Supplementary material for: Dysregulation and prognostic potential of 5-methylcytosine (5mC), 5-hydroxymethylcytosine (5hmC), 5-formylcytosine (5fC), and 5-carboxylcytosine (5caC) levels in prostate cancer
Source: Clin Epigenetics. 2018 Aug 7;10:105. doi: 10.1186/s13148-018-0540-x (PMC6081903; doi:10.1186/s13148-018-0540-x)
Supplement: Supplementary file 13 — Table S4. 5mC score (continuous and dichotomized) in univariate Cox regression analysis of BCR-free survival. Significant p values are highlighted in bold. (DOCX 16 kb) [file 13148_2018_540_MOESM13_ESM.docx]

**Additional file 13: Table S4.**

**5mC score (continuous and dichotomized) in univariate Cox regression analysis of BCR-free survival**

|  | **Full PC patient set**  **(n=344, 152 BCR)** | | | ***ERG-* PC patient subset  (n=150, 67 BCR)** | | | ***ERG+* PC patient set (n=192, 85 BCR)** | | |
| --- | --- | --- | --- | --- | --- | --- | --- | --- | --- |
|  | **Univariate** | | | **Univariate** | | | **Univariate** | | |
| **Variable** | **HR  (95% CI)** | **p-value** | **C-index** | **HR  (95% CI)** | **p-value** | **C-index** | **HR  (95% CI)** | **p-value** | **C-index** |
| **5mC score (continuous)** | 1.04  (0.72-1.50) | 0.834 | 0.51 | 1.26  (0.69-2.28) | 0.454 | 0.54 | 0.88  (0.54-1.43) | 0.599 | 0.52 |
| **5mC score (dichotomized)** | 1.18  (0.85-1.62) | 0.318 | 0.53 | 1.23  (0.76-2.00) | 0.401 | 0.54 | 1.08  (0.70-1.69) | 0.723 | 0.51 |
| **Pre-op. PSA  (≤10 *vs.* >10 ng/ml)** | 2.62  (1.82-3.79) | **<0.001** | 0.62 | 2.57  (1.40-4.72) | **0.002** | 0.59 | 2.82  (1.76-4.50) | **<0.001** | 0.65 |
| **Gleason score  (<7 *vs.* ≥7)** | 2.11  (1.48-3.00) | **<0.001** | 0.59 | 1.76  (1.03-3.02) | **0.040** | 0.57 | 2.43  (1.52-3.90) | **<0.001** | 0.61 |
| **Surgical margin  (neg. *vs.* pos.)** | 1.30  (1.18-1.44) | **<0.001** | 0.63 | 2.57  (1.59-4.17) | **<0.001** | 0.61 | 1.25  (1.12-1.40) | **<0.001** | 0.64 |
| **Tumor stage  (≤ pT2c *vs.* ≥pT3a)** | 3.05  (2.21-4.21) | **<0.001** | 0.64 | 2.33  (1.44-3.76) | **0.001** | 0.61 | 3.75  (2.42-5.80) | **<0.001** | 0.66 |
| ***ERG* status  (neg. *vs.* pos.)** | 1.03  (0.75-1.42) | 0.840 | 0.51 | - | - | - | - | - | - |

Significant p-values are highlighted in bold.
