# Supplementary material for: Dysregulation and prognostic potential of 5-methylcytosine (5mC), 5-hydroxymethylcytosine (5hmC), 5-formylcytosine (5fC), and 5-carboxylcytosine (5caC) levels in prostate cancer
Source: Clin Epigenetics. 2018 Aug 7;10:105. doi: 10.1186/s13148-018-0540-x (PMC6081903; doi:10.1186/s13148-018-0540-x)
Supplement: Supplementary file 14 — Table S5. 5hmC score (continuous) in univariate and multivariate Cox regression analysis of BCR-free survival. (DOCX 18 kb) [file 13148_2018_540_MOESM14_ESM.docx]

**Additional file 14: Table S5.**

**5hmC score (continuous) in univariate and multivariate Cox regression analysis of BCR-free survival**

| **Full PC patient set (n=367, 158 BCR)** | | | | | | | |
| --- | --- | --- | --- | --- | --- | --- | --- |
|  | **Univariate** | | | **Multivariate^a^** | | | |
| **Variable** | **HR (95% CI)** | **p-value** | **C-index** | **HR (95% CI)** | **p-value** | **C-index^b^** | **C-index^c^** |
| **5hmC score (continuous)** | 1.37 (1.01-1.87) | **0.045** | 0.55 | 1.44 (1.04-1.98) | **0.026** | 0.75 | - |
| **Pre-op. PSA  (≤10 *vs.* >10 ng/ml)** | 2.78 (1.94-3.98) | **<0.001** | 0.63 | 2.33 (1.62-3.37) | **<0.001** |  | 0.74 |
| **Gleason score  (<7 *vs.* ≥7)** | 2.25 (1.59-3.19) | **<0.001** | 0.60 | 1.95 (1.37-2.79) | **<0.001** |  |  |
| **Surgical margin  (neg. *vs.* pos.)** | 2.98 (2.17-4.09) | **<0.001** | 0.64 | 2.11 (1.51-2.95) | **<0.001** |  |  |
| **Tumor stage  (≤ pT2c *vs.* ≥pT3a)** | 3.01 (2.20-4.13) | **<0.001** | 0.63 | 1.98 (1.41-2.77) | **<0.001** |  |  |
| ***ERG* status  (neg. *vs.* pos.)** | 1.15 (0.84-1.58) | 0.386 | 0.52 | - | - |  |  |
| ***ERG-* PC patient subset (n=161, 66 BCR)** | | | | | | | |
|  | **Univariate** | | | **Multivariate^a^** | | | |
| **Variable** | **HR (95% CI)** | **p-value** | **C-index** | **HR (95% CI)** | **p-value** | **C-index^b^** | **C-index^c^** |
| **5hmC score  (continuous)** | 1.62 (1.02-2.57) | **0.043** | 0.59 | 1.69 (1.02-2.82) | **0.042** | 0.75 | - |
| **Pre-op. PSA  (≤10 *vs.* >10 ng/ml)** | 2.65 (1.46-4.78) | **0.001** | 0.60 | 2.65 (1.46-4.83) | **0.001** |  | 0.73 |
| **Gleason score  (<7 *vs.* ≥7)** | 2.38 (1.36-4.15) | **0.002** | 0.60 | 1.89 (1.07-3.33) | **0.027** |  |  |
| **Surgical margin  (neg. *vs.* pos.)** | 3.06 (1.88-4.96) | **<0.001** | 0.63 | 2.21 (1.30-3.77) | **0.004** |  |  |
| **Tumor stage  (≤ pT2c *vs.* ≥pT3a)** | 2.86 (1.76-4.64) | **<0.001** | 0.62 | 1.81 (1.05-3.10) | **0.032** |  |  |
| ***ERG+* PC patient subset (n=206, 92 BCR)** | | | | | | | |
|  | **Univariate** | | | **Multivariate^a^** | | | |
| **Variable** | **HR (95% CI)** | **p-value** | **C-index** | **HR (95% CI)** | **p-value** | **C-index^b^** | **C-index^c^** |
| **5hmC score  (continuous)** | 1.14 (0.75-1.75) | 0.537 | 0.51 | - | **-** | - | - |
| **Pre-op. PSA  (≤10 *vs.* >10 ng/ml)** | 3.03 (1.92-4.77) | **<0.001** | 0.65 | 2.17 (1.33-3.53) | **0.002** | - | 0.75 |
| **Gleason score  (<7 *vs.* ≥7)** | 2.19 (1.40-3.42) | **0.001** | 0.60 | 2.14 (1.34-3.40) | **0.001** | - |  |
| **Surgical margin  (neg. *vs.* pos.)** | 3.01 (1.98-4.60) | **<0.001** | 0.64 | 2.20 (1.42-3.42) | **<0.001** | - |  |
| **Tumor stage  (≤ pT2c *vs.* ≥pT3a)** | 3.15 (2.08-4.77) | **<0.001** | 0.64 | 1.99 (1.26-3.13) | **0.003** | - |  |

^a^ Final multivariate model including only significant variables. ^b^ Harrell’s C-index for final model including 5hmC. ^c^ Harrell’s C-index for final model excluding 5hmC. Significant p-values are highlighted in bold.
