# Supplementary material for: Dysregulation and prognostic potential of 5-methylcytosine (5mC), 5-hydroxymethylcytosine (5hmC), 5-formylcytosine (5fC), and 5-carboxylcytosine (5caC) levels in prostate cancer
Source: Clin Epigenetics. 2018 Aug 7;10:105. doi: 10.1186/s13148-018-0540-x (PMC6081903; doi:10.1186/s13148-018-0540-x)
Supplement: Supplementary file 15 — Table S6. 5hmC score (dichotomized) in univariate and multivariate Cox regression analysis of BCR-free survival. (DOCX 19 kb) [file 13148_2018_540_MOESM15_ESM.docx]

**Additional file 15: Table S6.**

**5hmC score (dichotomized) in univariate and multivariate Cox regression analysis of BCR-free survival**

| **Full PC patient set (n=367, 158 BCR)** | | | | | | | | | |
| --- | --- | --- | --- | --- | --- | --- | --- | --- | --- |
|  | **Univariate** | | | **Multivariate^b^** | | | | | |
| **Variable** | **HR  (95% CI)** | **p-value** | **C-index** | **HR  (95% CI)** | **p-value** | **C-index^c^** | **C-index^d^** |  | |
| **5hmC score  (dichotomized)** | 1.40  (1.02-1.92) | **0.038** | 0.55 | 1.40  (1.02-1.94) | **0.039** | 0.75 | - |  | |
| **Pre-op. PSA  (≤10 *vs.* >10 ng/ml)** | 2.78  (1.94-3.98) | **<0.001** | 0.63 | 2.32  (1.61-3.5) | **<0.001** |  | 0.74 |  |  |
| **Gleason score  (<7 *vs.* ≥7)** | 2.25  (1.59-3.19) | **<0.001** | 0.60 | 1.96  (1.37-2.80) | **<0.001** |  |  |  |  |
| **Surgical margin  (neg. *vs.* pos.)** | 2.98  (2.17-4.09) | **<0.001** | 0.64 | 2.15  (1.54-3.01) | **<0.001** |  |  |  |  |
| **Tumor stage  (≤ pT2c *vs.* ≥pT3a)** | 3.01  (2.20-4.13) | **<0.001** | 0.63 | 1.94  (1.38-2.72) | **<0.001** |  |  |  |  |
| ***ERG* status  (neg. *vs.* pos.)** | 1.15  (0.84-1.58) | 0.386 | 0.52 | - | - |  |  |  |  |
| ***ERG*- PC patient subset (n=161, 66 BCR)** | | | | | | | | | |
|  | **Univariate** | | | **Multivariate^a^** | | **Multivariate^b^** | | | |
| **Variable** | **HR  (95% CI)** | **p-value** | **C-index** | **HR  (95% CI)** | **p-value** | **HR  (95% CI)** | **p- value** | **C-index^c^** | **C-index^d^** |
| **5hmC score  (dichotomized)** | 1.93  (1.19-3.14) | **0.008** | 0.60 | 1.69  (1.02-2.82) | **0.042** | 2.10  (1.29-3.43) | **0.003** | 0.75 | - |
| **Pre-op. PSA  (≤10 *vs.* >10 ng/ml)** | 2.65  (1.46-4.78) | **0.001** | 0.60 | 2.65  (1.46-4.83) | **0.001** | 2.56  (1.41-4.64) | **0.002** |  | 0.73 |
| **Gleason score  (<7 *vs.* ≥7)** | 2.38  (1.36-4.15) | **0.002** | 0.60 | 1.89  (1.07-3.33) | **0.027** | 1.88  (1.07-3.30) | **0.027** |  |  |
| **Surgical margin  (neg. *vs.* pos.)** | 3.06  (1.88-4.96) | **<0.001** | 0.63 | 2.21  (1.30-3.77) | **0.004** | 2.85  (1.74-4.66) | **<0.001** |  |  |
| **Tumor stage  (≤ pT2c *vs.* ≥pT3a)** | 2.86  (1.76-4.64) | **<0.001** | 0.62 | 1.81  (1.05-3.10) | **0.032** | **-** | **-** |  |  |
| ***ERG+* PC patient subset (n=206, 92 BCR)** | | | | | | | | | |
|  | **Univariate** | | | **Multivariate^b^** | | | | | |
| **Variable** | **HR  (95% CI)** | **p-value** | **C-index** | **HR  (95% CI)** | **p-value** | **C-index^c^** | | | |
| **5hmC score  (dichotomized)** | 1.05  (0.69-1.61) | 0.805 | 0.50 | - | **-** | - | | | |
| **Pre-op. PSA  (≤10 *vs.* >10 ng/ml)** | 3.03  (1.92-4.77) | **<0.001** | 0.65 | 2.17 (1.33-3.53) | **0.002** | 0.75 | | | |
| **Gleason score  (<7 *vs.* ≥7)** | 2.19  (1.40-3.42) | **0.001** | 0.60 | 2.14  (1.34-3.40) | **0.001** |  |  |  |  |
| **Surgical margin  (neg. *vs.* pos.)** | 3.01  (1.98-4.60) | **<0.001** | 0.64 | 2.20  (1.42-3.42) | **<0.001** |  |  |  |  |
| **Tumor stage  (≤ pT2c *vs.* ≥pT3a)** | 3.15  (2.08-4.77) | **<0.001** | 0.64 | 1.99 (1.26-3.13) | **0.003** |  |  |  |  |

^a^ Global multivariate model including all parameters. ^b^ Final multivariate model including only significant variables. ^c^ Harrell’s C-index for final model including 5hmC. ^d^ Harrell’s C-index for final model excluding 5hmC. Significant p-values are highlighted in bold.
