# Supplementary material for: Dysregulation and prognostic potential of 5-methylcytosine (5mC), 5-hydroxymethylcytosine (5hmC), 5-formylcytosine (5fC), and 5-carboxylcytosine (5caC) levels in prostate cancer
Source: Clin Epigenetics. 2018 Aug 7;10:105. doi: 10.1186/s13148-018-0540-x (PMC6081903; doi:10.1186/s13148-018-0540-x)
Supplement: Supplementary file 16 — Table S7. 5fC score (continuous and dichotomized) in univariate Cox regression analysis of BCR-free survival. (DOCX 15 kb) [file 13148_2018_540_MOESM16_ESM.docx]

**Additional file 16: Table S7.**

**5fC score (continuous and dichotomized) in univariate Cox regression analysis of BCR-free survival**

|  | **Full PC patient set (n=281, 126 BCR)** | | | ***ERG-* PC patient subset (n= 135, 59 BCR)** | | | ***ERG+* PC patient subset (n=138, 65 BCR)** | | |
| --- | --- | --- | --- | --- | --- | --- | --- | --- | --- |
|  | **Univariate** | | | **Univariate** | | | **Univariate** | | |
| **Variable** | **HR  (95% CI)** | **p-value** | **C-index** | **HR  (95% CI)** | **p-value** | **C-index** | **HR  (95% CI)** | **p-value** | **C-index** |
| **5fC score  (continuous)** | 0.94  (0.73-1.20) | 0.613 | 0.51 | 1.10  (0.78-1.56) | 0.583 | 0.53 | 0.75  (0.52-1.09) | 0.130 | 0.55 |
| **5fC score (dichotomized)** | 1.02  (0.67-1.56) | 0.926 | 0.50 | 1.38  (0.77-2.49) | 0.282 | 0.54 | 0.67  (0.34-1.33) | 0.253 | 0.53 |
| **Pre-op. PSA  (≤10 *vs.* >10 ng/ml)** | 2.67  (1.76-4.05) | **<0.001** | 0.61 | 2.92  (1.47-5.77) | **0.002** | 0.60 | 2.71  (1.58-4.64) | **<0.001** | 0.63 |
| **Gleason score  (<7 *vs.* ≥7)** | 2.10  (1.43-3.09) | **<0.001** | 0.59 | 2.00  (1.13-3.54) | **0.018** | 0.58 | 2.22  (1.31-3.77) | **0.003** | 0.60 |
| **Surgical margin  (neg. *vs.* pos.)** | 2.74  (1.93-3.92) | **<0.001** | 0.62 | 2.21  (1.33-3.69) | **0.002** | 0.59 | 3.50  (2.11-5.80) | **<0.001** | 0.66 |
| **Tumor stage  (≤ pT2c *vs.* ≥pT3a)** | 3.15  (2.21-4.48) | **<0.001** | 0.63 | 2.34  (1.40-3.91) | **0.001** | 0.60 | 3.88  (2.35-6.42) | **<0.001** | 0.66 |
| ***ERG* status  (neg. *vs.* pos.)** | 1.15  (0.81-1.63) | 0.444 | 0.52 | - | - | - | - | - | - |

Significant p-values are highlighted in bold.
