# Supplementary material for: Dysregulation and prognostic potential of 5-methylcytosine (5mC), 5-hydroxymethylcytosine (5hmC), 5-formylcytosine (5fC), and 5-carboxylcytosine (5caC) levels in prostate cancer
Source: Clin Epigenetics. 2018 Aug 7;10:105. doi: 10.1186/s13148-018-0540-x (PMC6081903; doi:10.1186/s13148-018-0540-x)
Supplement: Supplementary file 17 — Table S8. 5caC score (continuous) in univariate and multivariate Cox regression analysis of BCR-free survival. (DOCX 16 kb) [file 13148_2018_540_MOESM17_ESM.docx]

**Additional file 17: Table S8.**

**5caC score (continuous) in univariate and multivariate Cox regression analysis of BCR-free survival**

| **Full PC patient set (n=351, 149 BCR)** | | | | | ***ERG-* PC patient subset (n=160, 65 BCR)** | | | |
| --- | --- | --- | --- | --- | --- | --- | --- | --- |
|  | **Univariate** | | | | **Univariate** | | | |
| **Variable** | **HR (95% CI)** | **p-value** | | **C-index** | **HR (95% CI)** | | **p-value** | **C-index** |
| **5caC score**  **(continuous)** | 0.91 (0.73-1.14) | 0.419 | | 0.53 | 1.21 (0.86-1.71) | | 0.277 | 0.54 |
| **Pre-op. PSA**  **(≤10 *vs.* >10 ng/ml)** | 2.81 (1.94-4.06) | **<0.001** | | 0.63 | 2.62 (1.45-4.73) | | **0.001** | 0.60 |
| **Gleason score  (<7 *vs.* ≥7)** | 2.51 (1.74-3.62) | **<0.001** | | 0.61 | 2.43 (1.38-4.28) | | **0.002** | 0.60 |
| **Surgical margin  (neg. *vs.* pos.)** | 1.37 (1.23-1.52) | **<0.001** | | 0.64 | 2.99 (1.83-4.88) | | **<0.001** | 0.63 |
| **Tumor stage**  **(≤ pT2c *vs.* ≥pT3a)** | 3.42 (2.47-4.73) | **<0.001** | | 0.65 | 2.80 (1.72-4.57) | | **<0.001** | 0.62 |
| ***ERG* status  (neg. *vs.* pos.)** | 1.18 (0.85-1.63) | 0.316 | | 0.53 | - | | - | - |
| ***ERG+* PC patient subset (n=183, 81 BCR)** | | | | | | | | |
|  | **Univariate** | | | | **Multivariate** | | | |
| **Variable** | **HR (95% CI)** | **p-value** | **C-index** | | **HR (95% CI)** | **p-value** | | |
| **5caC score (continuous)** | 0.68 (0.51-0.92) | **0.011** | 0.60 | | 0.84 (0.61-1.17) | 0.299 | | |
| **Pre-op. PSA  (≤10 *vs.* >10 ng/ml)** | 3.04 (1.89-4.90) | **<0.001** | 0.65 | | 1.88 (1.11-3.16) | **0.018** | | |
| **Gleason score  (<7 *vs.* ≥7)** | 2.61 (1.61-4.24) | **<0.001** | 0.62 | | 2.40 (1.46-3.94) | **0.001** | | |
| **Surgical margin  (neg. *vs.* pos.)** | 1.29 (1.14-1.47) | **<0.001** | 0.65 | | 2.05 (1.28-3.28) | **0.003** | | |
| **Tumor stage  (≤ pT2c *vs.* ≥pT3a)** | 4.02 (2.58-6.25) | **<0.001** | 0.67 | | 2.52 (1.54-4.13) | **<0.001** | | |

Significant p-values are highlighted in bold.
