# Supplementary material for: Dysregulation and prognostic potential of 5-methylcytosine (5mC), 5-hydroxymethylcytosine (5hmC), 5-formylcytosine (5fC), and 5-carboxylcytosine (5caC) levels in prostate cancer
Source: Clin Epigenetics. 2018 Aug 7;10:105. doi: 10.1186/s13148-018-0540-x (PMC6081903; doi:10.1186/s13148-018-0540-x)
Supplement: Supplementary file 18 — Table S9. 5caC score (dichotomized) in univariate and multivariate Cox regression analysis of BCR-free survival. (DOCX 20 kb) [file 13148_2018_540_MOESM18_ESM.docx]

**Additional file 18: Table S9.**

**5caC score (dichotomized) in univariate and multivariate Cox regression analysis of BCR-free survival**

| **Full PC patient set (n=351, 149 BCR)** | | | | ***ERG*- PC patient subset (n=160, 65 BCR)** | | |
| --- | --- | --- | --- | --- | --- | --- |
|  | **Univariate** | | | **Univariate** | | |
| **Variable** | **HR (95% CI)** | **p-value** | **C-index** | **HR (95% CI)** | **p-value** | **C-index** |
| **5caC score  (dichotomized)** | 1.01 (0.73-1.40) | 0.966 | 0.49 | 1.61 (0.98-2.63) | 0.058 | 0.56 |
| **Pre-op. PSA  (≤10 *vs.* >10 ng/ml)** | 2.81 (1.94-4.06) | **<0.001** | 0.63 | 2.62 (1.45-4.73) | **0.001** | 0.60 |
| **Gleason score  (<7 *vs.* ≥7)** | 2.51 (1.74-3.62) | **<0.001** | 0.61 | 2.43 (1.38-4.28) | **0.002** | 0.60 |
| **Surgical margin  (neg. *vs.* pos.)** | 1.37 (1.23-1.52) | **<0.001** | 0.64 | 2.99 (1.83-4.88) | **<0.001** | 0.63 |
| **Tumor stage  (≤ pT2c *vs.* ≥pT3a)** | 3.42 (2.47-4.73) | **<0.001** | 0.65 | 2.80 (1.72-4.57) | **<0.001** | 0.62 |
| ***ERG* status  (neg. *vs.* pos.)** | 1.18 (0.85-1.63) | 0.316 | 0.53 | - | - | - |
| ***ERG+* PC patient subset (n=183, 81 BCR)** | | | | | | |
|  | **Univariate** | | | **Multivariate^a^** | | |
| **Variable** | **HR (95% CI)** | **p-value** | **C-index** | **HR (95% CI)** | **p-value** | |
| **5caC score  (dichotomized)** | 0.62 (0.40-0.97) | **0.034** | 0.57 | 0.73 (0.46-1.16) | 0.182 | |
| **Pre-op. PSA  (≤10 *vs.* >10 ng/ml)** | 3.04 (1.89-4.90) | **<0.001** | 0.65 | 1.85 (1.10-3.11) | **0.021** | |
| **Gleason score  (<7 *vs.* ≥7)** | 2.61 (1.61-4.24) | **<0.001** | 0.62 | 2.48 (1.50-4.09) | **<0.001** | |
| **Surgical margin  (neg. *vs.* pos.)** | 1.29 (1.14-1.47) | **<0.001** | 0.65 | 2.12 (1.31-3.41) | **0.002** | |
| **Tumor stage  (≤ pT2c *vs.* ≥pT3a)** | 4.02 (2.58-6.25) | **<0.001** | 0.67 | 2.52 (1.54-4.12) | **<0.001** | |

^a^ Final multivariate model including only significant variables. Significant p-values are highlighted in bold.
